# Two new Rb–Ga arsenates: RbGa(HAsO_4_)_2_ and RbGa_2_As(HAsO_4_)_6_

**DOI:** 10.1107/S2056989018011180

**Published:** 2018-08-14

**Authors:** Karolina Schwendtner, Uwe Kolitsch

**Affiliations:** a Institute for Chemical Technology and Analytics, Division of Structural Chemistry, TU Wien, Getreidemarkt 9/164-SC, 1060 Vienna, Austria; b Naturhistorisches Museum Wien, Burgring 7, 1010 Wien, and Institut für Mineralogie und Kristallographie, Universität Wien, Althanstrasse 14, 1090 Wien, Austria

**Keywords:** RbGa(HAsO_4_)_2_, RbGa_2_As(HAsO_4_)_6_, AsO_6_, arsenate, hydrogenarsenate, AsO_6_ octa­hedra, crystal structure

## Abstract

The crystal structures of hydro­thermally synthesized (*T* = 493 K, 7–9 d) rubidium gallium bis­[hydrogenarsenate(V)], RbGa(HAsO_4_)_2_, and rubidium digallium arsenic(V) hexa­[hydrogenarsenate(V)], RbGa_2_As(HAsO_4_)_6_, were solved by single-crystal X-ray diffraction. Both compounds have tetra­hedral–octa­hedral framework topologies. The *M* ^+^ cations are located in channels of the respective framework. RbGa(HAsO_4_)_2_ crystallizes in the RbFe(HPO_4_)_2_ structure type (*R*  *c*), while RbGa_2_As(HAsO_4_)_6_ adopts the structure type of RbAl_2_As(HAsO_4_)_6_ (*R*  *c*), which represents a modification of the RbFe(HPO_4_)_2_ structure type. In this modification, one third of the *M* ^3+^O_6_ octa­hedra are replaced by AsO_6_ octa­hedra, and two thirds of the voids in the structure, which are usually filled by *M* ^+^ cations, remain empty to achieve charge balance.

## Abstract 2

The crystal structures of hydro­thermally synthesized RbGa(HAsO_4_)_2_ and RbGa_2_As(HAsO_4_)_6_ were solved by single-crystal X-ray diffraction. They both crystallize in related *R* 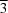 *c* structure types, one of which contains AsO_6_ octa­hedra assuming the topological role of *M* ^3+^O_6_ octa­hedra.

## Chemical context   

Compounds with mixed tetra­hedral–octa­hedral (T–O) framework structures feature a broad range of different atomic arrangements, resulting in topologies with various inter­esting properties, such as ion exchange (Masquelier *et al.*, 1996 ▸) and ion conductivity (Chouchene *et al.*, 2017 ▸), as well as unusual piezoelectric (Ren *et al.*, 2015 ▸), magnetic (Ouerfelli *et al.*, 2007 ▸) or nonlinear optical features (frequency doubling) (Sun *et al.*, 2017 ▸). In order to further increase the insufficient knowledge about the crystal chemistry and structure types of arsenates, a comprehensive study of the system *M* ^+^–*M* ^3+^–O–(H)–As^5+^ (*M* ^+^ = Li, Na, K, Rb, Cs, Ag, Tl, NH_4_; *M* ^3+^ = Al, Ga, In, Sc, Fe, Cr, Tl) was undertaken, which led to a large number of new compounds, most of which have been published (Schwendtner & Kolitsch, 2007 ▸, 2017 ▸, 2018*a* ▸,*b* ▸, and references therein).

Among the many different structure types found during our study, one atomic arrangement, *i.e.* the RbFe(HPO_4_)_2_ type (Lii & Wu, 1994 ▸; rhombohedral, *R* 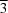 *c*), was found to show a large crystal–chemical flexibility which allows the incorporation of a wide variety of cations. A total of nine representatives of this structure type are presently known among *M* ^+^ *M* ^3+^(H*T*O_4_)_2_ (*T* = P, As) compounds containing Rb or Cs as the *M* ^+^ cation and Al, Ga, Fe or In as the *M* ^3+^ cation (Lesage *et al.*, 2007 ▸; Lii & Wu, 1994 ▸; Schwendtner & Kolitsch, 2017 ▸, 2018*a* ▸,*b* ▸), including RbGa(HPO_4_)_2_ (Lesage *et al.*, 2007 ▸). One of the title compounds, RbGa(HAsO_4_)_2_, is another new representative of the RbFe(HPO_4_)_2_ structure type. The second title compound, RbGa_2_As(HAsO_4_)_6_, is the third representative of a recently described variation of the RbFe(HPO_4_)_2_ type, the RbAl_2_As(HAsO_4_)_6_ type. It also crystallizes in *R* 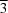 *c* and up to now members with RbAl and CsFe as *M* ^+^ *M* ^3+^ cation combinations are known (Schwendtner & Kolitsch, 2018*b* ▸). Inter­estingly, all presently known *M* ^+^ *M* ^3+^ combinations adopting this new structure type also have representatives adopting the RbFe(HPO_4_)_2_ type. It thus seems likely that more of the known RbFe(HPO_4_)_2_-type arsenates would also adopt the new RbAl_2_As(HAsO_4_)_6_-type atomic arrangement under formally ‘dry’ synthesis conditions (see §3). RbGa_2_As(HAsO_4_)_6_ is a rare example of a compound containing AsO_6_ octa­hedra. Out of all reported arsenates(V), only about 3% contain AsO_6_ polyhedra, according to our earlier review paper (Schwendtner & Kolitsch, 2007 ▸), which provides an overview of all known compounds containing AsO_6_ groups and their bond-length statistics. At present, 37 compounds containing As in an octa­hedral coordination are known (Schwendtner & Kolitsch, 2018*b* ▸); RbGa_2_As(HAsO_4_)_6_ represents the 38^th^ member of this class of compounds. While 12 Rb- and Ga-containing phosphates are contained in the ICSD (FIZ, 2018 ▸), only one Rb–Ga arsenate, *i.e.* RbGaF_3_(H_2_AsO_4_) (Marshall *et al.*, 2015 ▸), is known so far. Since submitting this paper, another paper dealing with isotypic *M* ^+^ *M* ^3+^_2_As(HAsO_4_)_6_ compounds (*M* ^+^ *M* ^3+^ = TlGa, CsGa, CsAl) has been published (Schwendtner & Kolitsch, 2018*c* ▸).

## Structural commentary   

**Figure 1:**
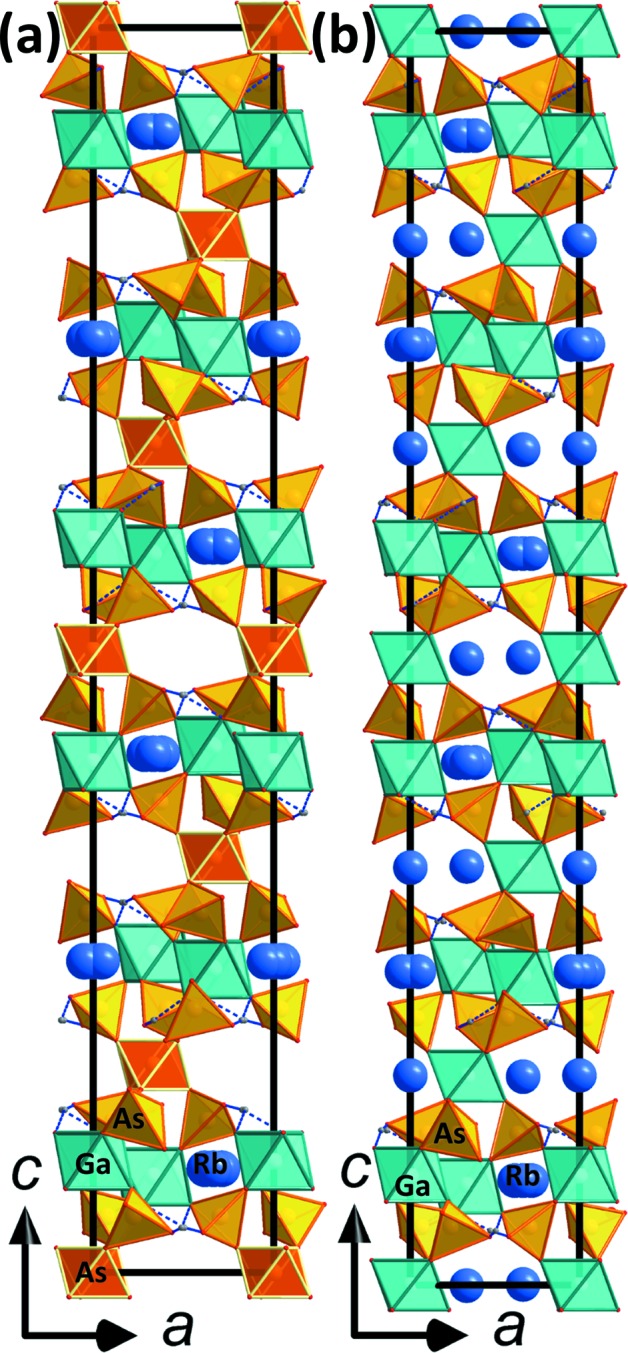
Crystal structure drawings of (*a*) RbGa_2_As(HAsO_4_)_6_ and (*b*) RbGa(HAsO_4_)_2_ in views along the *b* axis. A part of the GaO_6_ octa­hedra is replaced by AsO_6_ octa­hedra in RbGa_2_As(HAsO_4_)_6_; the corresponding layers (see Figs. 2 ▸ and 3 ▸) are compressed along *c* and the corresponding void remains vacant of Rb atoms.

**Figure 2:**
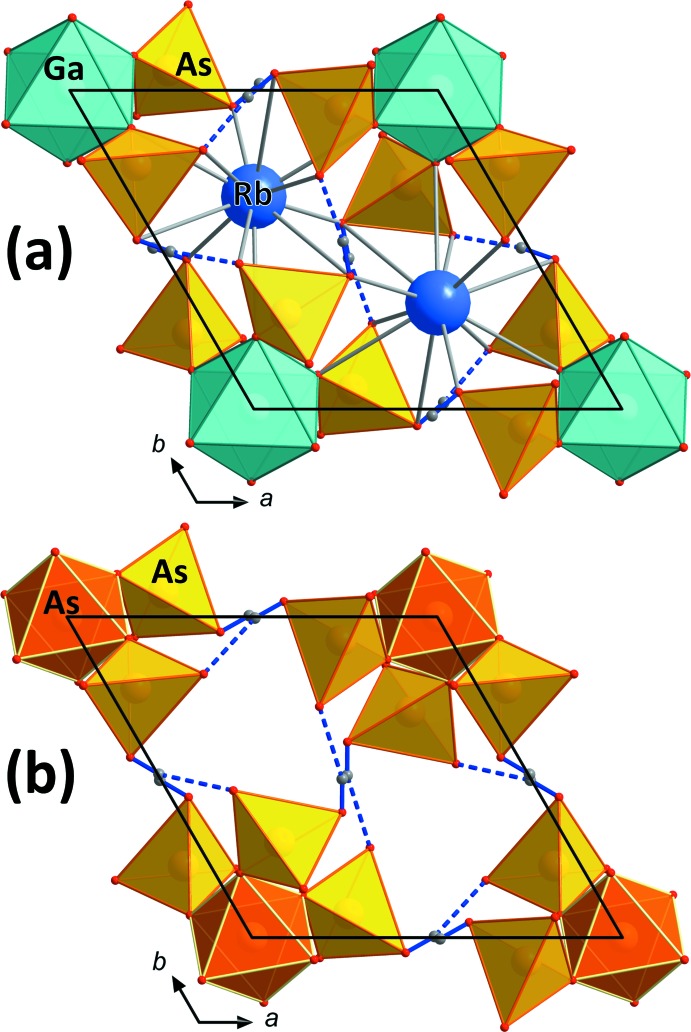
Crystal structure drawings of (*a*) RbGa(HAsO_4_)_2_ and (*b*) RbGa_2_As(HAsO_4_)_6_ inequal layers, viewed along the *c* axis. In this layer, the GaO_6_ octa­hedra are replaced by AsO_6_ octa­hedra in RbGa_2_As(HAsO_4_)_6_ (*b*). Since the unit-cell dimensions in directions *a* and *b* are slightly longer in RbGa_2_As(HAsO_4_)_6_ and the AsO_6_ octa­hedra are smaller than the corresponding GaO_6_ octa­hedra, the (Ga/As)As_6_O_24_ units within this layer move further apart – leading to longer *D*—H⋯*A* distances and a compressed (along *c*) void that is too small for Rb atoms (compare Fig. 1 ▸).

**Figure 3:**
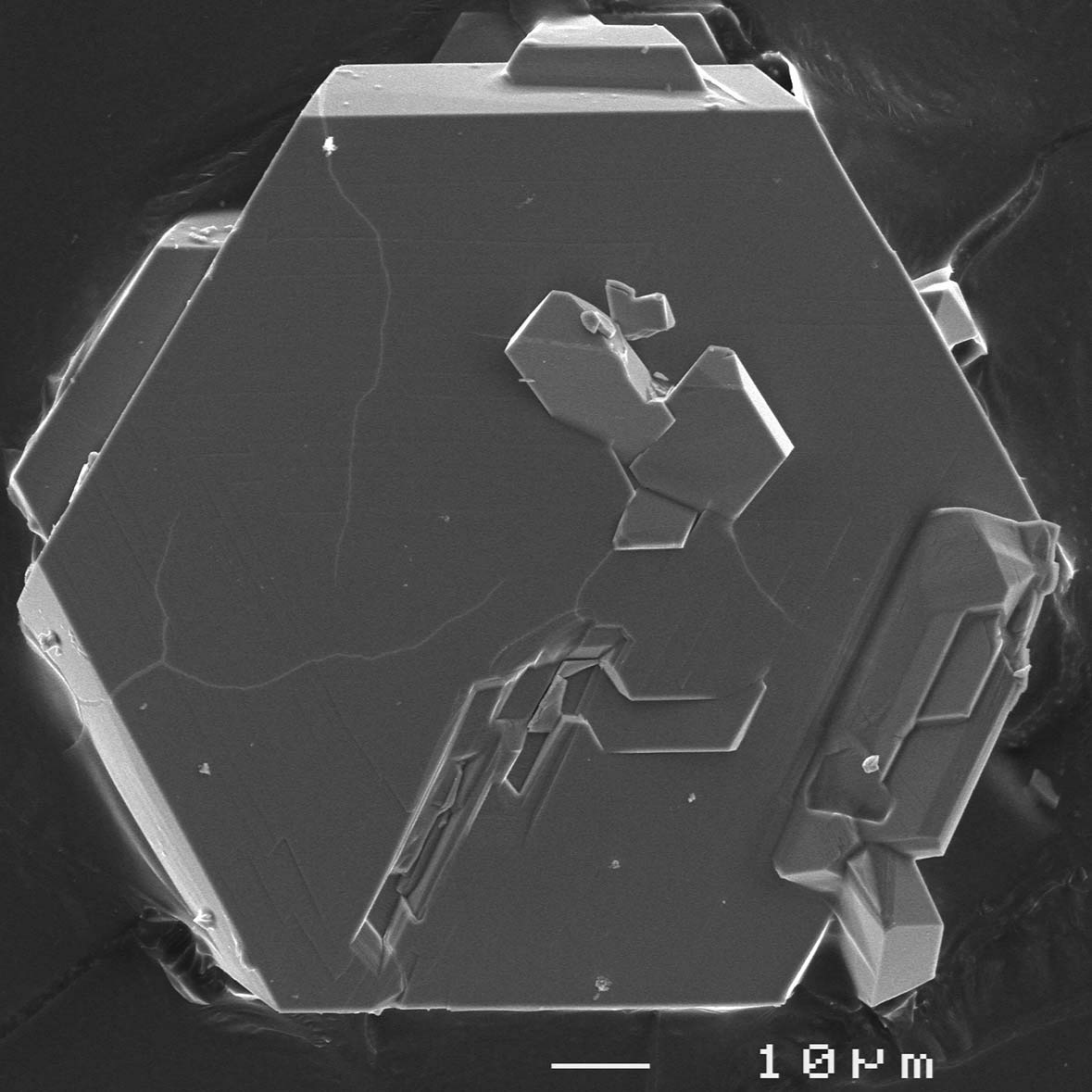
SEM micrograph of the pseudohexa­gonal tabular crystals of RbGa(HAsO_4_)_2_.

The two title compounds are very closely related to each other and are modifications of a basic tetra­hedral–octa­hedral framework structure featuring inter­penetrating channels, which host the *M* ^+^ cations (Fig. 1 ▸). The two structure types, first reported for RbFe(HPO_4_)_2_ (*R* 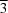 *c*; Lii & Wu, 1994 ▸) and RbAl_2_As(HAsO_4_)_6_ (*R* 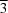 *c*; Schwendtner & Kolitsch, 2018*b* ▸), are also related to the triclinic (NH_4_)Fe(HPO_4_)_2_ type (*P* 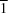; Yakubovich, 1993 ▸) and the RbAl(HAsO_4_)_2_ type (*R*32; Schwendtner & Kolitsch, 2018*b* ▸). The fundamental building unit in all these structure types contains *M* ^3+^O_6_ octa­hedra which are connected *via* their six corners to six protonated AsO_4_ tetra­hedra, thereby forming an *M* ^3+^As_6_O_24_ unit. These units are in turn connected *via* three corners to other *M* ^3+^O_6_ octa­hedra. The free protonated corner of each AsO_4_ tetra­hedron forms a hydrogen bond to the neighbouring *M* ^3+^As_6_O_24_ group (Fig. 2 ▸). The *M* ^3+^As_6_O_24_ units are arranged in layers perpendicular to the *c*_hex_ axis (Fig. 1 ▸). The units within these layers are held together by medium–strong hydrogen bonds (Tables 1 ▸ and 2 ▸). Both title compounds invariably show a very similar crystal habit: strongly pseudohexa­gonal to pseudo-octa­hedral (*cf.* Fig. 3 ▸).

**Figure 4:**
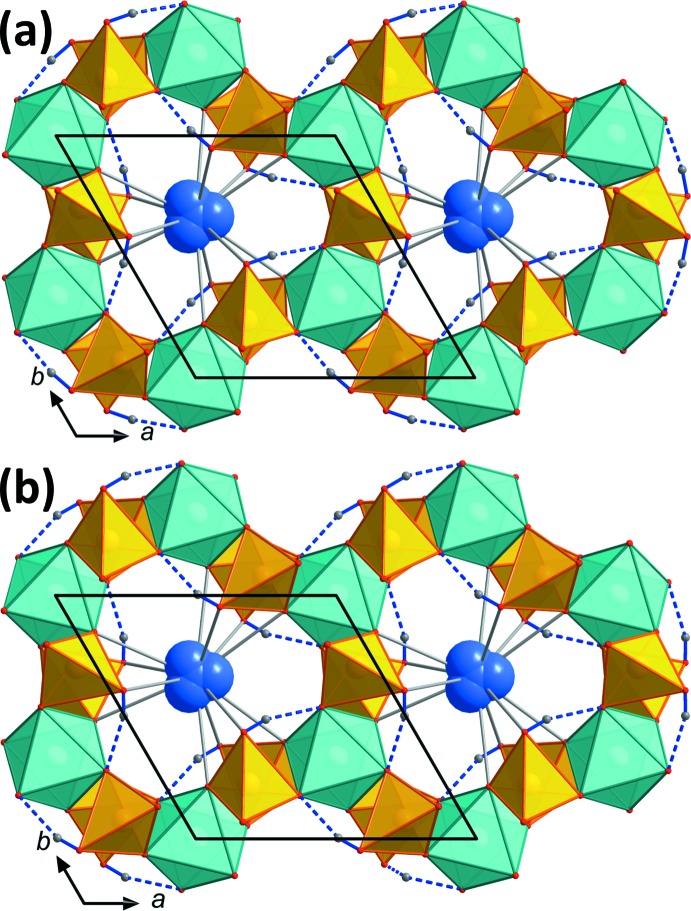
Crystal structure drawing of (*a*) RbGa(HAsO_4_)_2_ and (*b*) RbGa_2_As(HAsO_4_)_6_ equal layers, viewed along the *a* axis. In these topologically equivalent layers, there are no visible differences between the two structure types apart from very minor changes in the hydrogen-bond geometries. The Rb atoms in both compounds show a slight positional disorder and are 12-coordinated.

The new compound RbGa_2_As(HAsO_4_)_6_ could only be grown by ‘dry’ hydro­thermal techniques (without the addition of water). The extreme abundance of As during the synthesis and the formation of a melt of arsenic acid promotes the formation of this novel structure type and endorses the octa­hedral coordination of As. The substitution of one third of all Ga^3+^ cations by As^5+^ requires that two thirds of all Rb^+^ cations are omitted to achieve charge balance (compare Figs. 1 ▸ *a*, 1*b*, 2 ▸ *a* and 2*b*). This substitution also has an effect on the unit-cell parameters (Table 3 ▸) and the pore diameter. Since GaO_6_ is only replaced by AsO_6_ in every second layer (perpendicular to the *c* axis), the *a* axis must remain long enough to still be able to house the GaO_6_ in the layers between. The effect of the smaller AsO_6_ octa­hedra is therefore mainly reflected by a strong compression of about 5% along the *c* axis, while the *a* axis becomes even slightly longer compared to RbGa(HAsO_4_)_2_. Due to the comparatively smaller AsO_6_ octa­hedra, the (Ga/As)As_6_O_24_ units are further apart in RbGa_2_As(HAsO_4_)_6_ and the encased void is compressed along *c*, making it too small to house Rb^+^ cations (Figs. 1 ▸ and 2 ▸). This effect is also reflected by the considerably elongated hydrogen bond in RbGa_2_As(HAsO_4_)_6_. While these bonds, which connect neighbouring (Ga/As)As_6_O_24_ groups, are very strong in RbGa(HAsO_4_)_2_ [*D*—H⋯*A* = 2.598 (2) Å], they are much longer in RbGa_2_As(HAsO_4_)_6_ [2.7314 (17) Å; compare Tables 1 ▸ and 2 ▸]. The second layer, in contrast, remains practically identical in both compounds and contains Rb atoms with a slight positional disorder (Fig. 4 ▸). In both compounds, the Rb atoms are 12-coordinated (Figs. 2 ▸ and 3 ▸), and the average Rb—O bond lengths in RbGa_2_As(HAsO_4_)_6_ (3.433 Å) are longer than the longest average bond length in RbO_12_ polyhedra of 3.410 Å reported so far (Gagné & Hawthorne, 2016 ▸), thus leading to rather low bond-valence sums (BVSs; Gagné & Hawthorne, 2015 ▸) of only 0.59 valence units (v.u.), whereas the corresponding BVSs are 0.82 and 0.84 v.u. for RbGa(HAsO_4_)_2._ These loose bondings lead to considerable positional disorder of the Rb^+^ cations in these voids, which were modelled with two Rb positions, between 0.41 (2) and 0.42 (4) Å apart. While position Rb1*A* in the centre of the large framework void in RbGa_2_As(HAsO_4_)_6_ has only 77% occupancy compared to the off-centre position Rb1*B* (with occupancy 23%), in RbGa(HAsO_4_)_2_, the central position Rb1*A* has 91% occupancy. Similar behaviour was observed for the isotypic CsFe and RbAl compounds (Schwendtner & Kolitsch, 2018*b* ▸), as well as isotypic phosphates (Lesage *et al.*, 2007 ▸).

A further indirect effect of the substituting AsO_6_ octa­hedra is a distinct change in the As—O distances of the AsO_4_ tetra­hedra. The average As—O distance in the protonated AsO_4_ tetra­hedra, with values between 1.688 and 1.689 Å, is in both compounds very close to the statistical average of 1.686 (10) Å (Schwendtner, 2008 ▸). Also the BVSs (Gagné & Hawthorne, 2015 ▸) are close to ideal values (4.98–5.00 v.u.). In RbGa(HAsO_4_)_2_, the HAsO_4_ tetra­hedra show a typical distortion, with three short As—O distances to attached GaO_6_ octa­hedra and one elongated As—O bond length for the protonated O atom involved in the O—H bond. That bond length (Table 4 ▸) in RbGa(HAsO_4_)_2_ is slightly longer [1.7417 (17) Å] than the average distance of As—O⋯H bonds in HAsO_4_ groups [1.72 (3) Å; Schwendtner, 2008 ▸]. In contrast, RbGa_2_As(HAsO_4_)_6_ has two short ^[4]^As—O bond lengths to neighbouring GaO_6_ octa­hedra, but the ^[4]^As—O bond length of the O atom shared with the AsO_6_ octa­hedra is also elongated [1.7100 (11) Å] due to ^[4]^As—O—^[6]^As repulsion. The ^[4]^As—OH bond is therefore shortened to 1.7122 (13) Å (Table 5 ▸). The average As—O distances in the AsO_6_ octa­hedra are the shortest average distances of AsO_6_ octa­hedra found so far, *i.e.* 1.807 Å, leading to rather high BVSs of 5.33 v.u. (after Gagné & Hawthorne, 2015 ▸). The grand mean As—O bond distance in AsO_6_ octa­hedra in inorganic compounds is 1.830 (2) Å according to Schwendtner & Kolitsch (2007 ▸ *a*); this value was determined on 33 AsO_6_ octa­hedra of 31 compounds. Gagné & Hawthorne (2018 ▸) determined an identical, but less precise, value of 1.830 (28) Å, based on only 13 AsO_6_ octa­hedra in AsO_6_-containing compounds meeting all selection criteria as defined in Gagné & Hawthorne (2016 ▸). However, a larger number of compounds meeting these criteria were not used by Gagné & Hawthorne (2018 ▸) for unknown reasons. The average Ga—O bond lengths of the octa­hedrally coordinated Ga cations (1.962–1.964 Å) are slightly shorter than the grand mean average of 1.978 (17) Å (Gagné & Hawthorne, 2018 ▸), explaining the corresponding BVSs of 3.10 to 3.11 v.u.

## Synthesis and crystallization   

The compounds were grown by hydro­thermal synthesis at 493 K (7 d, autogeneous pressure, slow furnace cooling) using Teflon-lined stainless steel autoclaves with an approximate filling volume of 2 ml. Reagent-grade Rb_2_CO_3_, Ga_2_O_3_ and H_3_AsO_4_·0.5H_2_O were used as starting reagents in approximate volume ratios of Rb:Ga:As of 1:1:3 for both synthesis batches. For RbGa(HAsO_4_)_2_ the vessels were filled with distilled water to about 70% of their inner volumes, which led to initial and final pH values of 1.5. The reaction product was washed thoroughly with distilled water, filtered and dried at room temperature. RbGa(HAsO_4_)_2_ formed colourless pseudohexa­gonal platelets (Fig. 3 ▸) and is stable in air.

**Figure 5:**
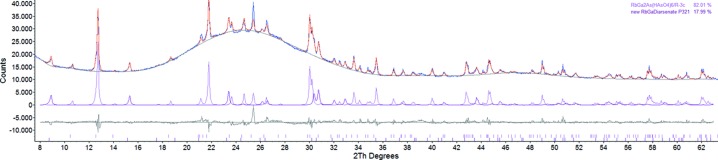
Graph of the Rietveld refinement (*TOPAS*; Bruker, 2009 ▸) of RbGa_2_As(HAsO_4_)_6_, showing the partial alteration of the pseudo-octa­hedral crystals after an 11-year storage in air. The crystals were hygroscopic and had partly transformed to an amorphous mass. The presence of the relics of the unaltered primary crystals are still visible (pink curve), but a newly crystallized overgrowth of extremely fine fibrous crystals could be attributed to a new strongly protonated Rb–Ga diarsenate with space group *P*321 (dark-red curve), which will be the subject of a future publication.

For RbGa_2_As(HAsO_4_)_6_, which contains As in both tetra­hedral and octa­hedral coordination, no additional water was added and arsenic acid was present in excess to promote the growth of crystals from a melt or even vapour of arsenic acid under extremely acidic conditions. RbGa_2_As(HAsO_4_)_6_ formed large colourless pseudo-octa­hedral crystals accompanied by small colourless twinned crystals of RbH_3_As_4_O_12_ (Schwendtner & Kolitsch, 2007 ▸). The crystals of RbGa_2_As(HAsO_4_)_6_ were extracted mechanically and not further washed; they are hygroscopic and decompose slowly over a period of several years to an amorphous gel and a new, strongly protonated diarsenate containing Rb and Ga (*P*321, publication in preparation). This slow partial alteration is illustrated in an X-ray powder diffraction pattern (Fig. 5 ▸).

A measured X-ray powder diffraction diagram of RbGa(HAsO_4_)_2_ was deposited at the Inter­national Centre for Diffraction Data under PDF number 00-057-0239 (Wohl­schlaeger *et al.*, 2006 ▸).

## Experimental and refinement   

Crystal data, data collection and structure refinement are given in Table 3 ▸. For the refinement of RbGa(HAsO_4_)_2_, the coordinates of RbFe(HPO_4_)_2_ (Lii & Wu, 1994 ▸) were used for the final refinement steps. H atoms were then located from difference Fourier maps and added to the model. For the refinement of RbGa_2_As(HAsO_4_)_6_, the model for RbAl_2_As(HAsO_4_)_6_ (Schwendtner & Kolitsch, 2018*b* ▸) was used as a starting point. In both compounds, O—H bonds were restrained to 0.9±0.04 Å. During the last refinement steps, residual electron-density peaks of up to 3.83 and 1.16 e Å^−3^ were located 0.63 and 0.68 Å from the Rb sites in RbGa_2_As(HAsO_4_)_6_ and RbGa(HAsO_4_)_2_, respectively, suggesting irregular displacement parameters and split positions, similar to what was found for RbFe(HPO_4_)_2_-type RbAl(HPO_4_)_2_ (Lesage *et al.*, 2007 ▸). Therefore, a further position, Rb1*B*, was included in both refinements, which refined to low occupancies and led to considerable decreases in the *R* factors and weight parameters for both compounds. The bulk occupancies of Rb1*A* + Rb1*B* were constrained to give a total occupancy of 1.00. The final residual electron densities in both compounds are < 1 e Å^−3^.

## Supplementary Material

Crystal structure: contains datablock(s) RbGa2AsHAsO46, RbGaHAsO42, global. DOI: 10.1107/S2056989018011180/ff2155sup1.cif

Structure factors: contains datablock(s) RbGa2AsHAsO46. DOI: 10.1107/S2056989018011180/ff2155RbGa2AsHAsO46sup2.hkl

Structure factors: contains datablock(s) RbGaHAsO42. DOI: 10.1107/S2056989018011180/ff2155RbGaHAsO42sup3.hkl

CCDC references: 1860366, 1860365

Additional supporting information:  crystallographic information; 3D view; checkCIF report

## Figures and Tables

**Table 1:** Hydrogen-bond geometry (Å, °) for RbGa(HAsO_4_)_2_

*D*—H⋯*A*	*D*—H	H⋯*A*	*D*⋯*A*	*D*—H⋯*A*
O3—H⋯O4^xxi^	0.85 (3)	1.76 (3)	2.598 (2)	168 (4)

Symmetry code: (xxi) 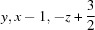.

**Table 2:** Hydrogen-bond geometry (Å, °) for RbGa_2_As(HAsO_4_)_6_

*D*—H⋯*A*	*D*—H	H⋯*A*	*D*⋯*A*	*D*—H⋯*A*
O3—H⋯O4^xiv^	0.80 (3)	1.98 (3)	2.7314 (17)	158 (3)

Symmetry code: (xiv) 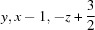.

**Table 3:** Experimental details

	**RbGa(HAsO_4_)_2_**	**RbGa_2_As(HAsO_4_)_6_**
Crystal data		
Chemical formula	RbGa(HAsO_4_)_2_	RbGa_2_As(HAsO_4_)_6_
*M*_r_	435.05	1139.40
Crystal system, space group	Trigonal, *R* 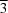 *c*:*H*	Trigonal, *R* 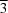 *c*:*H*
Temperature (K)	293	293
*a*, *c* (Å)	8.385 (1), 53.880 (11)	8.491 (1), 50.697 (11)
*V* (Å^3^)	3280.7 (10)	3165.4 (10)
*Z*	18	6
Radiation type	Mo *K*α	Mo *K*α
μ (mm^−1^)	19.42	15.85
Crystal size (mm)	0.07 × 0.07 × 0.02	0.13 × 0.12 × 0.12

Data collection		
Diffractometer	Nonius KappaCCD single-crystal four-circle	Nonius KappaCCD single-crystal four-circle
Absorption correction	Multi-scan (*SCALEPACK*; Otwinowski *et al.*, 2003 ▸)	Multi-scan (*SCALEPACK*; Otwinowski *et al.*, 2003 ▸)
*T*_min_, *T*_max_	0.343, 0.697	0.232, 0.252
No. of measured, independent and observed [*I* > 2σ(*I*)] reflections	3896, 1079, 1027	4684, 1287, 1196
*R*_int_	0.016	0.016
(sin θ/λ)_max_ (Å^−1^)	0.704	0.757

Refinement		
*R*[*F* ^2^ > 2σ(*F* ^2^)], *wR*(*F* ^2^), *S*	0.016, 0.040, 1.11	0.014, 0.034, 1.13
No. of reflections	1079	1287
No. of parameters	68	65
No. of restraints	2	2
H-atom treatment	All H-atom parameters refined	All H-atom parameters refined
Δρ_max_, Δρ_min_ (e Å^−3^)	0.79, −0.53	0.75, −0.84

Computer programs: *COLLECT* (Nonius, 2003 ▸), *DENZO* and *SCALEPACK* (Otwinowski *et al.*, 2003 ▸), *SHELXS97* (Sheldrick, 2008 ▸), *SHELXL2016* (Sheldrick, 2015 ▸), *DIAMOND* (Brandenburg, 2005 ▸) and *publCIF* (Westrip, 2010 ▸).

**Table 4:** Selected bond lengths (Å) for RbGa(HAsO_4_)_2_

Rb1*A*—O3^i^	3.197 (2)	Rb2—O4^xi^	3.4960 (16)
Rb1*A*—O3^ii^	3.197 (2)	Rb2—O3^xii^	3.5327 (19)
Rb1*A*—O3^iii^	3.197 (2)	Rb2—O3^xiii^	3.533 (2)
Rb1*A*—O3^iv^	3.197 (2)	Rb2—O3^xiv^	3.5328 (19)
Rb1*A*—O3^v^	3.197 (2)	Ga1—O2^xv^	1.9596 (14)
Rb1*A*—O3	3.197 (2)	Ga1—O2^iii^	1.9597 (15)
Rb1*A*—O2^ii^	3.3698 (16)	Ga1—O2^xvi^	1.9597 (15)
Rb1*A*—O2^iii^	3.3698 (16)	Ga1—O4^xvii^	1.9690 (15)
Rb1*A*—O2^v^	3.3699 (16)	Ga1—O4^iv^	1.9690 (15)
Rb1*A*—O2	3.3699 (16)	Ga1—O4^xviii^	1.9690 (15)
Rb1*A*—O2^i^	3.3699 (15)	Ga2—O1^viii^	1.9625 (15)
Rb1*A*—O2^iv^	3.3699 (15)	Ga2—O1^xiv^	1.9625 (16)
Rb2—O3	2.9346 (17)	Ga2—O1^xix^	1.9625 (15)
Rb2—O3^iv^	2.9347 (17)	Ga2—O1^iv^	1.9626 (15)
Rb2—O3^iii^	2.9347 (17)	Ga2—O1^xviii^	1.9626 (16)
Rb2—O1^vi^	3.3714 (16)	Ga2—O1^xvii^	1.9626 (15)
Rb2—O1^vii^	3.3715 (16)	As—O1^xx^	1.6576 (15)
Rb2—O1^viii^	3.3715 (17)	As—O2	1.6724 (15)
Rb2—O4^ix^	3.4960 (16)	As—O4^ii^	1.6805 (15)
Rb2—O4^x^	3.4960 (17)	As—O3	1.7417 (17)

Symmetry codes: (i) 
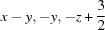
; (ii) 
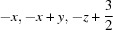
; (iii) 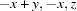; (iv) 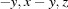; (v) 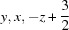; (vi) 
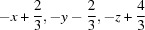
; (vii) 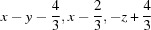; (viii) 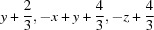; (ix) 
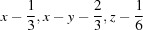
; (x) 
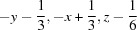
; (xi) 
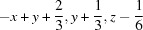
; (xii) 
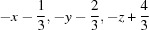
; (xiii) 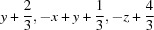; (xiv) 
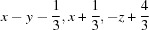
; (xv) 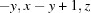; (xvi) 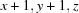; (xvii) 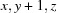; (xviii) 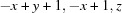; (xix) 
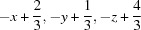
; (xx) 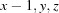.

**Table 5:** Selected bond lengths (Å) for RbGa_2_As(HAsO_4_)_6_

Rb1*A*—O3^i^	3.4212 (16)	Ga1—O4^vii^	1.9623 (11)
Rb1*A*—O3^ii^	3.4212 (16)	Ga1—O2^viii^	1.9625 (11)
Rb1*A*—O3^iii^	3.4212 (15)	Ga1—O2^iii^	1.9625 (11)
Rb1*A*—O3^iv^	3.4212 (16)	Ga1—O2^ix^	1.9625 (11)
Rb1*A*—O3^v^	3.4212 (15)	As1—O1^x^	1.8067 (11)
Rb1*A*—O3	3.4213 (16)	As1—O1^xi^	1.8068 (11)
Rb1*A*—O2^ii^	3.4438 (12)	As1—O1^xii^	1.8068 (11)
Rb1*A*—O2^iii^	3.4438 (12)	As1—O1^v^	1.8068 (11)
Rb1*A*—O2	3.4438 (12)	As1—O1^vii^	1.8068 (11)
Rb1*A*—O2^iv^	3.4438 (12)	As1—O1^vi^	1.8068 (11)
Rb1*A*—O2^i^	3.4438 (12)	As2—O2	1.6658 (11)
Rb1*A*—O2^v^	3.4438 (12)	As2—O4^ii^	1.6670 (11)
Ga1—O4^vi^	1.9623 (12)	As2—O1^xiii^	1.7100 (11)
Ga1—O4^v^	1.9623 (11)	As2—O3	1.7122 (13)

Symmetry codes: (i) 
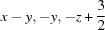
; (ii) 
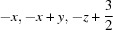
; (iii) 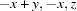; (iv) 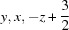; (v) 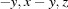; (vi) 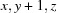; (vii) 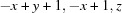; (viii) 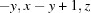; (ix) 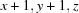; (x) 
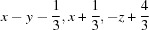
; (xi) 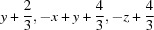; (xii) 
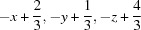
; (xiii) 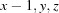.

## supplementary crystallographic information

### Rubidium digallium arsenic(V) hexakis[hydrogen arsenate(V)] (RbGa2AsHAsO46) Crystal data

**Table:**

RbGa_2_As(HAsO_4_)_6_	*D*_x_ = 3.586 Mg m^−^^3^
*M**_r_* = 1139.40	Mo *K*α radiation, λ = 0.71073 Å
Trigonal, *R*3*c*:*H*	Cell parameters from 2570 reflections
*a* = 8.491 (1) Å	θ = 2.0–32.6°
*c* = 50.697 (11) Å	µ = 15.85 mm^−^^1^
*V* = 3165.4 (10) Å^3^	*T* = 293 K
*Z* = 6	Large pseudo-octahedra, colourless
*F*(000) = 3168	0.13 × 0.12 × 0.12 mm

### Rubidium digallium arsenic(V) hexakis[hydrogen arsenate(V)] (RbGa2AsHAsO46) Data collection

**Table:**

Nonius KappaCCD single-crystal four-circle diffractometer	1196 reflections with *I* > 2σ(*I*)
Radiation source: fine-focus sealed tube	*R*_int_ = 0.016
φ and ω scans	θ_max_ = 32.5°, θ_min_ = 2.4°
Absorption correction: multi-scan (SCALEPACK; Otwinowski *et al.*, 2003)	*h* = −12→12
*T*_min_ = 0.232, *T*_max_ = 0.252	*k* = −10→10
4684 measured reflections	*l* = −75→76
1287 independent reflections	

### Rubidium digallium arsenic(V) hexakis[hydrogen arsenate(V)] (RbGa2AsHAsO46) Refinement

**Table:**

Refinement on *F*^2^	Secondary atom site location: difference Fourier map
Least-squares matrix: full	Hydrogen site location: difference Fourier map
*R*[*F*^2^ > 2σ(*F*^2^)] = 0.014	All H-atom parameters refined
*wR*(*F*^2^) = 0.034	*w* = 1/[σ^2^(*F*_o_^2^) + (0.0136*P*)^2^ + 10.4877*P*] where *P* = (*F*_o_^2^ + 2*F*_c_^2^)/3
*S* = 1.13	(Δ/σ)_max_ = 0.008
1287 reflections	Δρ_max_ = 0.75 e Å^−^^3^
65 parameters	Δρ_min_ = −0.83 e Å^−^^3^
2 restraints	Extinction correction: SHELXL2016 (Sheldrick, 2015), Fc^*^=kFc[1+0.001xFc^2^λ^3^/sin(2θ)]^-1/4^
Primary atom site location: structure-invariant direct methods	Extinction coefficient: 0.000271 (17)

### Rubidium digallium arsenic(V) hexakis[hydrogen arsenate(V)] (RbGa2AsHAsO46) Special details

**Table:**

Geometry. All esds (except the esd in the dihedral angle between two l.s. planes) are estimated using the full covariance matrix. The cell esds are taken into account individually in the estimation of esds in distances, angles and torsion angles; correlations between esds in cell parameters are only used when they are defined by crystal symmetry. An approximate (isotropic) treatment of cell esds is used for estimating esds involving l.s. planes.

### Rubidium digallium arsenic(V) hexakis[hydrogen arsenate(V)] (RbGa2AsHAsO46) Fractional atomic coordinates and isotropic or equivalent isotropic displacement parameters (Å^2^)

**Table:**

	*x*	*y*	*z*	*U*_iso_*/*U*_eq_	Occ. (<1)
Rb1A	0.000000	0.000000	0.750000	0.055 (3)	0.669 (3)
Rb1B	0.000000	−0.048 (2)	0.750000	0.0359 (11)	0.1103 (9)
Ga1	0.333333	0.666667	0.75604 (2)	0.00736 (6)	
As1	0.333333	0.666667	0.666667	0.00627 (7)	
As2	−0.44400 (2)	−0.40772 (2)	0.71144 (2)	0.00735 (5)	
O1	0.40249 (16)	−0.46597 (15)	0.68629 (2)	0.01016 (19)	
O2	−0.45254 (15)	−0.27043 (15)	0.73416 (2)	0.01025 (19)	
O3	−0.22887 (17)	−0.28592 (18)	0.69874 (3)	0.0194 (3)	
O4	0.48817 (15)	−0.12495 (15)	0.77869 (2)	0.0108 (2)	
H	−0.176 (4)	−0.337 (4)	0.7027 (6)	0.046 (9)*	

### Rubidium digallium arsenic(V) hexakis[hydrogen arsenate(V)] (RbGa2AsHAsO46) Atomic displacement parameters (Å^2^)

**Table:**

	*U*^11^	*U*^22^	*U*^33^	*U*^12^	*U*^13^	*U*^23^
Rb1A	0.050 (4)	0.050 (4)	0.066 (2)	0.0251 (18)	0.000	0.000
Rb1B	0.024 (4)	0.034 (4)	0.046 (4)	0.012 (2)	0.0044 (14)	0.0022 (7)
Ga1	0.00807 (8)	0.00807 (8)	0.00596 (12)	0.00403 (4)	0.000	0.000
As1	0.00747 (10)	0.00747 (10)	0.00389 (14)	0.00373 (5)	0.000	0.000
As2	0.00844 (7)	0.00803 (7)	0.00670 (7)	0.00495 (5)	0.00010 (5)	0.00053 (5)
O1	0.0138 (5)	0.0110 (5)	0.0078 (4)	0.0077 (4)	−0.0031 (4)	0.0000 (4)
O2	0.0109 (5)	0.0099 (5)	0.0098 (5)	0.0051 (4)	0.0005 (4)	−0.0022 (4)
O3	0.0121 (5)	0.0203 (6)	0.0264 (7)	0.0084 (5)	0.0076 (5)	0.0075 (5)
O4	0.0124 (5)	0.0095 (5)	0.0125 (5)	0.0070 (4)	−0.0030 (4)	−0.0042 (4)

### Rubidium digallium arsenic(V) hexakis[hydrogen arsenate(V)] (RbGa2AsHAsO46) Geometric parameters (Å, º)

**Table:**

Rb1A—Rb1B^i^	0.41 (2)	Rb1B—O2	3.4234 (12)
Rb1A—Rb1B^ii^	0.41 (2)	Rb1B—O2^iv^	3.4234 (12)
Rb1A—O3^iii^	3.4212 (16)	Rb1B—O3^iii^	3.694 (14)
Rb1A—O3^iv^	3.4212 (16)	Rb1B—O3^ii^	3.694 (14)
Rb1A—O3^ii^	3.4212 (15)	Rb1B—O2^ii^	3.810 (18)
Rb1A—O3^v^	3.4212 (16)	Rb1B—O2^iii^	3.810 (18)
Rb1A—O3^i^	3.4212 (15)	Rb1B—O4^vi^	3.819 (18)
Rb1A—O3	3.4213 (16)	Rb1B—O4^vii^	3.819 (18)
Rb1A—O2^iv^	3.4438 (12)	Rb1B—As2^v^	3.934 (8)
Rb1A—O2^ii^	3.4438 (12)	Rb1B—As2^i^	3.934 (8)
Rb1A—O2	3.4438 (12)	Ga1—O4^viii^	1.9623 (12)
Rb1A—O2^v^	3.4438 (12)	Ga1—O4^i^	1.9623 (11)
Rb1A—O2^iii^	3.4438 (12)	Ga1—O4^ix^	1.9623 (11)
Rb1A—O2^i^	3.4438 (12)	Ga1—O2^x^	1.9625 (11)
Rb1A—As2^iv^	4.1192 (5)	Ga1—O2^ii^	1.9625 (11)
Rb1A—As2^iii^	4.1192 (5)	Ga1—O2^xi^	1.9625 (11)
Rb1A—As2^v^	4.1192 (5)	As1—O1^xii^	1.8067 (11)
Rb1A—As2^ii^	4.1192 (4)	As1—O1^xiii^	1.8068 (11)
Rb1B—Rb1B^i^	0.70 (3)	As1—O1^xiv^	1.8068 (11)
Rb1B—Rb1B^ii^	0.70 (3)	As1—O1^i^	1.8068 (11)
Rb1B—O2^v^	3.136 (14)	As1—O1^ix^	1.8068 (11)
Rb1B—O2^i^	3.136 (14)	As1—O1^viii^	1.8068 (11)
Rb1B—O3^iv^	3.269 (6)	As2—O2	1.6658 (11)
Rb1B—O3	3.269 (6)	As2—O4^iv^	1.6670 (11)
Rb1B—O3^v^	3.358 (2)	As2—O1^xv^	1.7100 (11)
Rb1B—O3^i^	3.358 (2)	As2—O3	1.7122 (13)
			
Rb1B^i^—Rb1A—Rb1B^ii^	120.00 (7)	Rb1B^ii^—Rb1B—O4^vii^	153.31 (7)
Rb1B^i^—Rb1A—O3^iii^	64.81 (2)	O2^v^—Rb1B—O4^vii^	53.4 (3)
Rb1B^ii^—Rb1A—O3^iii^	77.69 (5)	O2^i^—Rb1B—O4^vii^	45.1 (2)
Rb1B^i^—Rb1A—O3^iv^	77.69 (2)	O3^iv^—Rb1B—O4^vii^	44.49 (19)
Rb1B^ii^—Rb1A—O3^iv^	129.70 (3)	O3—Rb1B—O4^vii^	98.0 (5)
O3^iii^—Rb1A—O3^iv^	68.57 (4)	O3^v^—Rb1B—O4^vii^	76.7 (3)
Rb1B^i^—Rb1A—O3^ii^	77.69 (2)	O3^i^—Rb1B—O4^vii^	93.3 (3)
Rb1B^ii^—Rb1A—O3^ii^	64.81 (5)	O2—Rb1B—O4^vii^	109.4 (4)
O3^iii^—Rb1A—O3^ii^	100.59 (5)	O2^iv^—Rb1B—O4^vii^	71.6 (2)
O3^iv^—Rb1A—O3^ii^	155.38 (5)	O3^iii^—Rb1B—O4^vii^	109.34 (9)
Rb1B^i^—Rb1A—O3^v^	129.70 (2)	O3^ii^—Rb1B—O4^vii^	157.2 (3)
Rb1B^ii^—Rb1A—O3^v^	64.81 (5)	O2^ii^—Rb1B—O4^vii^	144.11 (3)
O3^iii^—Rb1A—O3^v^	68.57 (4)	O2^iii^—Rb1B—O4^vii^	144.95 (3)
O3^iv^—Rb1A—O3^v^	68.57 (4)	O4^vi^—Rb1B—O4^vii^	53.5 (3)
O3^ii^—Rb1A—O3^v^	129.62 (5)	Rb1B^i^—Rb1B—As2^v^	137.8 (2)
Rb1B^i^—Rb1A—O3^i^	64.81 (2)	Rb1B^ii^—Rb1B—As2^v^	88.8 (3)
Rb1B^ii^—Rb1A—O3^i^	129.70 (3)	O2^v^—Rb1B—As2^v^	24.03 (3)
O3^iii^—Rb1A—O3^i^	129.62 (5)	O2^i^—Rb1B—As2^v^	107.9 (5)
O3^iv^—Rb1A—O3^i^	100.59 (5)	O3^iv^—Rb1B—As2^v^	82.2 (2)
O3^ii^—Rb1A—O3^i^	68.57 (4)	O3—Rb1B—As2^v^	82.5 (2)
O3^v^—Rb1A—O3^i^	155.38 (4)	O3^v^—Rb1B—As2^v^	25.63 (7)
Rb1B^i^—Rb1A—O3	129.70 (2)	O3^i^—Rb1B—As2^v^	145.3 (5)
Rb1B^ii^—Rb1A—O3	77.69 (5)	O2—Rb1B—As2^v^	51.65 (8)
O3^iii^—Rb1A—O3	155.38 (4)	O2^iv^—Rb1B—As2^v^	128.9 (3)
O3^iv^—Rb1A—O3	129.62 (5)	O3^iii^—Rb1B—As2^v^	91.62 (8)
O3^ii^—Rb1A—O3	68.57 (4)	O3^ii^—Rb1B—As2^v^	123.66 (15)
O3^v^—Rb1A—O3	100.59 (5)	O2^ii^—Rb1B—As2^v^	138.7 (4)
O3^i^—Rb1A—O3	68.57 (4)	O2^iii^—Rb1B—As2^v^	90.34 (14)
Rb1B^i^—Rb1A—O2^iv^	38.519 (18)	O4^vi^—Rb1B—As2^v^	68.6 (3)
Rb1B^ii^—Rb1A—O2^iv^	153.03 (7)	O4^vii^—Rb1B—As2^v^	67.9 (3)
O3^iii^—Rb1A—O2^iv^	76.93 (3)	Rb1B^i^—Rb1B—As2^i^	88.8 (2)
O3^iv^—Rb1A—O2^iv^	45.66 (3)	Rb1B^ii^—Rb1B—As2^i^	137.8 (2)
O3^ii^—Rb1A—O2^iv^	111.42 (3)	O2^v^—Rb1B—As2^i^	107.9 (5)
O3^v^—Rb1A—O2^iv^	113.17 (3)	O2^i^—Rb1B—As2^i^	24.03 (3)
O3^i^—Rb1A—O2^iv^	63.90 (3)	O3^iv^—Rb1B—As2^i^	82.5 (2)
O3—Rb1A—O2^iv^	127.31 (3)	O3—Rb1B—As2^i^	82.2 (2)
Rb1B^i^—Rb1A—O2^ii^	38.519 (18)	O3^v^—Rb1B—As2^i^	145.3 (5)
Rb1B^ii^—Rb1A—O2^ii^	83.75 (7)	O3^i^—Rb1B—As2^i^	25.63 (7)
O3^iii^—Rb1A—O2^ii^	63.90 (3)	O2—Rb1B—As2^i^	128.9 (3)
O3^iv^—Rb1A—O2^ii^	111.42 (3)	O2^iv^—Rb1B—As2^i^	51.65 (8)
O3^ii^—Rb1A—O2^ii^	45.66 (3)	O3^iii^—Rb1B—As2^i^	123.66 (15)
O3^v^—Rb1A—O2^ii^	127.31 (3)	O3^ii^—Rb1B—As2^i^	91.62 (8)
O3^i^—Rb1A—O2^ii^	76.93 (3)	O2^ii^—Rb1B—As2^i^	90.34 (14)
O3—Rb1A—O2^ii^	113.17 (3)	O2^iii^—Rb1B—As2^i^	138.7 (4)
O2^iv^—Rb1A—O2^ii^	77.04 (4)	O4^vi^—Rb1B—As2^i^	67.9 (3)
Rb1B^i^—Rb1A—O2	153.035 (19)	O4^vii^—Rb1B—As2^i^	68.6 (3)
Rb1B^ii^—Rb1A—O2	38.52 (7)	As2^v^—Rb1B—As2^i^	131.0 (5)
O3^iii^—Rb1A—O2	111.42 (3)	O4^viii^—Ga1—O4^i^	89.24 (5)
O3^iv^—Rb1A—O2	127.31 (3)	O4^viii^—Ga1—O4^ix^	89.24 (5)
O3^ii^—Rb1A—O2	76.93 (3)	O4^i^—Ga1—O4^ix^	89.24 (5)
O3^v^—Rb1A—O2	63.90 (3)	O4^viii^—Ga1—O2^x^	91.13 (5)
O3^i^—Rb1A—O2	113.17 (3)	O4^i^—Ga1—O2^x^	88.49 (5)
O3—Rb1A—O2	45.67 (3)	O4^ix^—Ga1—O2^x^	177.69 (5)
O2^iv^—Rb1A—O2	167.50 (4)	O4^viii^—Ga1—O2^ii^	177.69 (5)
O2^ii^—Rb1A—O2	114.733 (13)	O4^i^—Ga1—O2^ii^	91.13 (5)
Rb1B^i^—Rb1A—O2^v^	153.03 (2)	O4^ix^—Ga1—O2^ii^	88.48 (5)
Rb1B^ii^—Rb1A—O2^v^	83.75 (8)	O2^x^—Ga1—O2^ii^	91.17 (5)
O3^iii^—Rb1A—O2^v^	113.17 (3)	O4^viii^—Ga1—O2^xi^	88.48 (5)
O3^iv^—Rb1A—O2^v^	76.93 (3)	O4^i^—Ga1—O2^xi^	177.69 (5)
O3^ii^—Rb1A—O2^v^	127.31 (3)	O4^ix^—Ga1—O2^xi^	91.12 (5)
O3^v^—Rb1A—O2^v^	45.66 (3)	O2^x^—Ga1—O2^xi^	91.16 (5)
O3^i^—Rb1A—O2^v^	111.42 (3)	O2^ii^—Ga1—O2^xi^	91.16 (5)
O3—Rb1A—O2^v^	63.91 (3)	O4^viii^—Ga1—Rb1B^viii^	82.77 (12)
O2^iv^—Rb1A—O2^v^	114.733 (13)	O4^i^—Ga1—Rb1B^viii^	55.65 (8)
O2^ii^—Rb1A—O2^v^	167.50 (4)	O4^ix^—Ga1—Rb1B^viii^	143.89 (7)
O2—Rb1A—O2^v^	53.93 (4)	O2^x^—Ga1—Rb1B^viii^	33.99 (5)
Rb1B^i^—Rb1A—O2^iii^	83.749 (19)	O2^ii^—Ga1—Rb1B^viii^	99.31 (13)
Rb1B^ii^—Rb1A—O2^iii^	38.52 (7)	O2^xi^—Ga1—Rb1B^viii^	123.61 (10)
O3^iii^—Rb1A—O2^iii^	45.66 (3)	O4^viii^—Ga1—Rb1B^i^	143.89 (6)
O3^iv^—Rb1A—O2^iii^	113.17 (3)	O4^i^—Ga1—Rb1B^i^	82.77 (12)
O3^ii^—Rb1A—O2^iii^	63.90 (3)	O4^ix^—Ga1—Rb1B^i^	55.65 (8)
O3^v^—Rb1A—O2^iii^	76.93 (3)	O2^x^—Ga1—Rb1B^i^	123.61 (9)
O3^i^—Rb1A—O2^iii^	127.31 (3)	O2^ii^—Ga1—Rb1B^i^	33.99 (5)
O3—Rb1A—O2^iii^	111.42 (3)	O2^xi^—Ga1—Rb1B^i^	99.31 (12)
O2^iv^—Rb1A—O2^iii^	114.732 (14)	Rb1B^viii^—Ga1—Rb1B^i^	119.554 (12)
O2^ii^—Rb1A—O2^iii^	53.93 (4)	O4^viii^—Ga1—Rb1B^ix^	55.65 (10)
O2—Rb1A—O2^iii^	77.04 (4)	O4^i^—Ga1—Rb1B^ix^	143.89 (7)
O2^v^—Rb1A—O2^iii^	114.731 (14)	O4^ix^—Ga1—Rb1B^ix^	82.77 (13)
Rb1B^i^—Rb1A—O2^i^	83.749 (19)	O2^x^—Ga1—Rb1B^ix^	99.31 (14)
Rb1B^ii^—Rb1A—O2^i^	153.03 (7)	O2^ii^—Ga1—Rb1B^ix^	123.61 (9)
O3^iii^—Rb1A—O2^i^	127.31 (3)	O2^xi^—Ga1—Rb1B^ix^	33.99 (6)
O3^iv^—Rb1A—O2^i^	63.90 (3)	Rb1B^viii^—Ga1—Rb1B^ix^	119.554 (7)
O3^ii^—Rb1A—O2^i^	113.17 (3)	Rb1B^i^—Ga1—Rb1B^ix^	119.554 (2)
O3^v^—Rb1A—O2^i^	111.42 (3)	O4^viii^—Ga1—Rb1A^viii^	80.57 (3)
O3^i^—Rb1A—O2^i^	45.66 (3)	O4^i^—Ga1—Rb1A^viii^	57.14 (3)
O3—Rb1A—O2^i^	76.93 (3)	O4^ix^—Ga1—Rb1A^viii^	144.67 (3)
O2^iv^—Rb1A—O2^i^	53.93 (4)	O2^x^—Ga1—Rb1A^viii^	33.28 (3)
O2^ii^—Rb1A—O2^i^	114.732 (13)	O2^ii^—Ga1—Rb1A^viii^	101.54 (3)
O2—Rb1A—O2^i^	114.732 (13)	O2^xi^—Ga1—Rb1A^viii^	122.02 (3)
O2^v^—Rb1A—O2^i^	77.04 (4)	Rb1B^viii^—Ga1—Rb1A^viii^	2.56 (14)
O2^iii^—Rb1A—O2^i^	167.50 (4)	Rb1B^i^—Ga1—Rb1A^viii^	122.12 (13)
Rb1B^i^—Rb1A—As2^iv^	60.329 (4)	Rb1B^ix^—Ga1—Rb1A^viii^	117.05 (14)
Rb1B^ii^—Rb1A—As2^iv^	151.382 (12)	O4^viii^—Ga1—Rb1A	144.67 (3)
O3^iii^—Rb1A—As2^iv^	77.70 (2)	O4^i^—Ga1—Rb1A	80.57 (3)
O3^iv^—Rb1A—As2^iv^	24.04 (2)	O4^ix^—Ga1—Rb1A	57.14 (3)
O3^ii^—Rb1A—As2^iv^	134.60 (2)	O2^x^—Ga1—Rb1A	122.02 (3)
O3^v^—Rb1A—As2^iv^	92.58 (3)	O2^ii^—Ga1—Rb1A	33.28 (3)
O3^i^—Rb1A—As2^iv^	77.98 (3)	O2^xi^—Ga1—Rb1A	101.53 (3)
O3—Rb1A—As2^iv^	125.97 (2)	Rb1B^viii^—Ga1—Rb1A	117.05 (14)
O2^iv^—Rb1A—As2^iv^	23.324 (18)	Rb1B^i^—Ga1—Rb1A	2.56 (13)
O2^ii^—Rb1A—As2^iv^	98.261 (18)	Rb1B^ix^—Ga1—Rb1A	122.12 (13)
O2—Rb1A—As2^iv^	146.607 (19)	Rb1A^viii^—Ga1—Rb1A	119.614 (1)
O2^v^—Rb1A—As2^iv^	92.721 (19)	O4^viii^—Ga1—Rb1A^xi^	57.14 (3)
O2^iii^—Rb1A—As2^iv^	122.596 (19)	O4^i^—Ga1—Rb1A^xi^	144.67 (4)
O2^i^—Rb1A—As2^iv^	49.72 (2)	O4^ix^—Ga1—Rb1A^xi^	80.57 (3)
Rb1B^i^—Rb1A—As2^iii^	67.492 (4)	O2^x^—Ga1—Rb1A^xi^	101.53 (3)
Rb1B^ii^—Rb1A—As2^iii^	60.33 (6)	O2^ii^—Ga1—Rb1A^xi^	122.02 (3)
O3^iii^—Rb1A—As2^iii^	24.04 (2)	O2^xi^—Ga1—Rb1A^xi^	33.28 (3)
O3^iv^—Rb1A—As2^iii^	92.58 (3)	Rb1B^viii^—Ga1—Rb1A^xi^	122.12 (14)
O3^ii^—Rb1A—As2^iii^	77.98 (3)	Rb1B^i^—Ga1—Rb1A^xi^	117.05 (13)
O3^v^—Rb1A—As2^iii^	77.70 (2)	Rb1B^ix^—Ga1—Rb1A^xi^	2.56 (14)
O3^i^—Rb1A—As2^iii^	125.97 (2)	Rb1A^viii^—Ga1—Rb1A^xi^	119.614 (1)
O3—Rb1A—As2^iii^	134.60 (2)	Rb1A—Ga1—Rb1A^xi^	119.614 (1)
O2^iv^—Rb1A—As2^iii^	92.720 (19)	O1^xii^—As1—O1^xiii^	92.59 (5)
O2^ii^—Rb1A—As2^iii^	49.72 (2)	O1^xii^—As1—O1^xiv^	92.59 (5)
O2—Rb1A—As2^iii^	98.261 (18)	O1^xiii^—As1—O1^xiv^	92.59 (5)
O2^v^—Rb1A—As2^iii^	122.595 (19)	O1^xii^—As1—O1^i^	87.41 (5)
O2^iii^—Rb1A—As2^iii^	23.323 (18)	O1^xiii^—As1—O1^i^	180.00 (9)
O2^i^—Rb1A—As2^iii^	146.608 (19)	O1^xiv^—As1—O1^i^	87.41 (5)
As2^iv^—Rb1A—As2^iii^	99.334 (9)	O1^xii^—As1—O1^ix^	180.0
Rb1B^i^—Rb1A—As2^v^	151.382 (7)	O1^xiii^—As1—O1^ix^	87.41 (5)
Rb1B^ii^—Rb1A—As2^v^	67.49 (6)	O1^xiv^—As1—O1^ix^	87.41 (5)
O3^iii^—Rb1A—As2^v^	92.58 (3)	O1^i^—As1—O1^ix^	92.58 (5)
O3^iv^—Rb1A—As2^v^	77.70 (2)	O1^xii^—As1—O1^viii^	87.42 (5)
O3^ii^—Rb1A—As2^v^	125.97 (2)	O1^xiii^—As1—O1^viii^	87.42 (5)
O3^v^—Rb1A—As2^v^	24.04 (2)	O1^xiv^—As1—O1^viii^	180.00 (6)
O3^i^—Rb1A—As2^v^	134.60 (2)	O1^i^—As1—O1^viii^	92.58 (5)
O3—Rb1A—As2^v^	77.98 (3)	O1^ix^—As1—O1^viii^	92.58 (5)
O2^iv^—Rb1A—As2^v^	122.596 (19)	O2—As2—O4^iv^	117.31 (6)
O2^ii^—Rb1A—As2^v^	146.608 (19)	O2—As2—O1^xv^	115.10 (6)
O2—Rb1A—As2^v^	49.72 (2)	O4^iv^—As2—O1^xv^	100.43 (5)
O2^v^—Rb1A—As2^v^	23.323 (17)	O2—As2—O3	104.11 (6)
O2^iii^—Rb1A—As2^v^	92.72 (2)	O4^iv^—As2—O3	111.03 (6)
O2^i^—Rb1A—As2^v^	98.261 (19)	O1^xv^—As2—O3	108.86 (6)
As2^iv^—Rb1A—As2^v^	99.334 (9)	O2—As2—Rb1B^ii^	50.1 (3)
As2^iii^—Rb1A—As2^v^	99.334 (9)	O4^iv^—As2—Rb1B^ii^	114.68 (16)
Rb1B^i^—Rb1A—As2^ii^	60.329 (3)	O1^xv^—As2—Rb1B^ii^	144.86 (16)
Rb1B^ii^—Rb1A—As2^ii^	67.49 (6)	O3—As2—Rb1B^ii^	58.0 (2)
O3^iii^—Rb1A—As2^ii^	77.98 (3)	O2—As2—Rb1B	58.6 (2)
O3^iv^—Rb1A—As2^ii^	134.60 (2)	O4^iv^—As2—Rb1B	106.7 (3)
O3^ii^—Rb1A—As2^ii^	24.04 (2)	O1^xv^—As2—Rb1B	151.7 (2)
O3^v^—Rb1A—As2^ii^	125.97 (2)	O3—As2—Rb1B	53.57 (7)
O3^i^—Rb1A—As2^ii^	77.70 (2)	Rb1B^ii^—As2—Rb1B	10.2 (5)
O3—Rb1A—As2^ii^	92.58 (2)	O2—As2—Rb1A	54.94 (4)
O2^iv^—Rb1A—As2^ii^	98.261 (18)	O4^iv^—As2—Rb1A	111.68 (4)
O2^ii^—Rb1A—As2^ii^	23.324 (18)	O1^xv^—As2—Rb1A	147.34 (4)
O2—Rb1A—As2^ii^	92.721 (19)	O3—As2—Rb1A	54.48 (5)
O2^v^—Rb1A—As2^ii^	146.607 (19)	Rb1B^ii^—As2—Rb1A	5.1 (3)
O2^iii^—Rb1A—As2^ii^	49.72 (2)	Rb1B—As2—Rb1A	5.4 (3)
O2^i^—Rb1A—As2^ii^	122.596 (19)	O2—As2—Rb1B^i^	56.09 (6)
As2^iv^—Rb1A—As2^ii^	120.658 (6)	O4^iv^—As2—Rb1B^i^	113.41 (9)
As2^iii^—Rb1A—As2^ii^	57.236 (12)	O1^xv^—As2—Rb1B^i^	145.26 (10)
As2^v^—Rb1A—As2^ii^	134.983 (5)	O3—As2—Rb1B^i^	52.42 (10)
Rb1B^i^—Rb1B—Rb1B^ii^	60.00 (2)	Rb1B^ii^—As2—Rb1B^i^	6.1 (3)
Rb1B^i^—Rb1B—O2^v^	161.6 (2)	Rb1B—As2—Rb1B^i^	6.7 (3)
Rb1B^ii^—Rb1B—O2^v^	108.4 (3)	Rb1A—As2—Rb1B^i^	2.49 (11)
Rb1B^i^—Rb1B—O2^i^	108.4 (2)	O2—As2—Rb1B^xvi^	93.36 (7)
Rb1B^ii^—Rb1B—O2^i^	161.60 (17)	O4^iv^—As2—Rb1B^xvi^	42.23 (4)
O2^v^—Rb1B—O2^i^	86.3 (5)	O1^xv^—As2—Rb1B^xvi^	80.78 (10)
Rb1B^i^—Rb1B—O3^iv^	91.2 (3)	O3—As2—Rb1B^xvi^	153.23 (5)
Rb1B^ii^—Rb1B—O3^iv^	122.4 (3)	Rb1B^ii^—As2—Rb1B^xvi^	126.58 (16)
O2^v^—Rb1B—O3^iv^	83.6 (3)	Rb1B—As2—Rb1B^xvi^	125.4 (2)
O2^i^—Rb1B—O3^iv^	69.1 (3)	Rb1A—As2—Rb1B^xvi^	127.56 (9)
Rb1B^i^—Rb1B—O3	122.4 (3)	Rb1B^i^—As2—Rb1B^xvi^	130.05 (2)
Rb1B^ii^—Rb1B—O3	91.2 (3)	O2—As2—Rb1A^xvii^	94.58 (4)
O2^v^—Rb1B—O3	69.1 (3)	O4^iv^—As2—Rb1A^xvii^	42.53 (4)
O2^i^—Rb1B—O3	83.6 (3)	O1^xv^—As2—Rb1A^xvii^	79.08 (4)
O3^iv^—Rb1B—O3	142.5 (7)	O3—As2—Rb1A^xvii^	153.36 (5)
Rb1B^i^—Rb1B—O3^v^	113.4 (3)	Rb1B^ii^—As2—Rb1A^xvii^	128.36 (6)
Rb1B^ii^—Rb1B—O3^v^	76.7 (3)	Rb1B—As2—Rb1A^xvii^	127.17 (12)
O2^v^—Rb1B—O3^v^	48.28 (11)	Rb1A—As2—Rb1A^xvii^	129.347 (10)
O2^i^—Rb1B—O3^v^	121.7 (5)	Rb1B^i^—As2—Rb1A^xvii^	131.84 (12)
O3^iv^—Rb1B—O3^v^	71.12 (11)	Rb1B^xvi^—As2—Rb1A^xvii^	1.79 (10)
O3—Rb1B—O3^v^	105.18 (19)	O2—As2—Rb1B^xviii^	92.74 (11)
Rb1B^i^—Rb1B—O3^i^	76.7 (3)	O4^iv^—As2—Rb1B^xviii^	46.4 (2)
Rb1B^ii^—Rb1B—O3^i^	113.4 (3)	O1^xv^—As2—Rb1B^xviii^	76.75 (13)
O2^v^—Rb1B—O3^i^	121.7 (5)	O3—As2—Rb1B^xviii^	157.05 (19)
O2^i^—Rb1B—O3^i^	48.28 (11)	Rb1B^ii^—As2—Rb1B^xviii^	129.10 (5)
O3^iv^—Rb1B—O3^i^	105.18 (19)	Rb1B—As2—Rb1B^xviii^	128.75 (3)
O3—Rb1B—O3^i^	71.12 (11)	Rb1A—As2—Rb1B^xviii^	130.52 (5)
O3^v^—Rb1B—O3^i^	169.0 (7)	Rb1B^i^—As2—Rb1B^xviii^	132.99 (17)
Rb1B^i^—Rb1B—O2	118.6 (3)	Rb1B^xvi^—As2—Rb1B^xviii^	4.8 (2)
Rb1B^ii^—Rb1B—O2	60.4 (4)	Rb1A^xvii^—As2—Rb1B^xviii^	3.88 (18)
O2^v^—Rb1B—O2	56.66 (12)	O2—As2—Rb1B^xvii^	97.48 (15)
O2^i^—Rb1B—O2	124.3 (5)	O4^iv^—As2—Rb1B^xvii^	39.02 (18)
O3^iv^—Rb1B—O2	133.6 (2)	O1^xv^—As2—Rb1B^xvii^	79.91 (7)
O3—Rb1B—O2	46.84 (5)	O3—As2—Rb1B^xvii^	149.72 (18)
O3^v^—Rb1B—O2	64.78 (4)	Rb1B^ii^—As2—Rb1B^xvii^	129.012 (18)
O3^i^—Rb1B—O2	115.33 (5)	Rb1B—As2—Rb1B^xvii^	127.02 (15)
Rb1B^i^—Rb1B—O2^iv^	60.4 (3)	Rb1A—As2—Rb1B^xvii^	129.587 (13)
Rb1B^ii^—Rb1B—O2^iv^	118.6 (3)	Rb1B^i^—As2—Rb1B^xvii^	132.07 (12)
O2^v^—Rb1B—O2^iv^	124.3 (5)	Rb1B^xvi^—As2—Rb1B^xvii^	4.2 (2)
O2^i^—Rb1B—O2^iv^	56.66 (12)	Rb1A^xvii^—As2—Rb1B^xvii^	3.66 (17)
O3^iv^—Rb1B—O2^iv^	46.84 (5)	Rb1B^xviii^—As2—Rb1B^xvii^	7.4 (3)
O3—Rb1B—O2^iv^	133.6 (2)	As2^xix^—O1—As1^xx^	131.96 (6)
O3^v^—Rb1B—O2^iv^	115.33 (5)	As2^xix^—O1—Rb1B^vii^	79.11 (18)
O3^i^—Rb1B—O2^iv^	64.78 (4)	As1^xx^—O1—Rb1B^vii^	129.23 (10)
O2—Rb1B—O2^iv^	179.0 (7)	As2—O2—Ga1^xvii^	123.99 (6)
Rb1B^i^—Rb1B—O3^iii^	48.36 (16)	As2—O2—Rb1B^ii^	105.9 (2)
Rb1B^ii^—Rb1B—O3^iii^	56.58 (15)	Ga1^xvii^—O2—Rb1B^ii^	125.54 (18)
O2^v^—Rb1B—O3^iii^	113.81 (3)	As2—O2—Rb1B	96.8 (3)
O2^i^—Rb1B—O3^iii^	128.32 (3)	Ga1^xvii^—O2—Rb1B	130.47 (10)
O3^iv^—Rb1B—O3^iii^	66.90 (12)	Rb1B^ii^—O2—Rb1B	11.2 (6)
O3—Rb1B—O3^iii^	147.5 (4)	As2—O2—Rb1A	101.74 (4)
O3^v^—Rb1B—O3^iii^	66.07 (15)	Ga1^xvii^—O2—Rb1A	128.51 (4)
O3^i^—Rb1B—O3^iii^	122.7 (4)	Rb1B^ii^—O2—Rb1A	4.6 (2)
O2—Rb1B—O3^iii^	105.6 (3)	Rb1B—O2—Rb1A	6.8 (3)
O2^iv^—Rb1B—O3^iii^	73.63 (18)	As2—O2—Rb1B^i^	102.64 (6)
Rb1B^i^—Rb1B—O3^ii^	56.58 (18)	Ga1^xvii^—O2—Rb1B^i^	128.80 (4)
Rb1B^ii^—Rb1B—O3^ii^	48.36 (15)	Rb1B^ii^—O2—Rb1B^i^	3.34 (18)
O2^v^—Rb1B—O3^ii^	128.32 (3)	Rb1B—O2—Rb1B^i^	9.3 (4)
O2^i^—Rb1B—O3^ii^	113.81 (3)	Rb1A—O2—Rb1B^i^	2.77 (12)
O3^iv^—Rb1B—O3^ii^	147.5 (4)	As2—O3—Rb1B	101.51 (6)
O3—Rb1B—O3^ii^	66.90 (12)	As2—O3—Rb1B^ii^	96.3 (3)
O3^v^—Rb1B—O3^ii^	122.7 (4)	Rb1B—O3—Rb1B^ii^	12.1 (6)
O3^i^—Rb1B—O3^ii^	66.07 (15)	As2—O3—Rb1A	101.48 (6)
O2—Rb1B—O3^ii^	73.63 (18)	Rb1B—O3—Rb1A	6.5 (3)
O2^iv^—Rb1B—O3^ii^	105.6 (3)	Rb1B^ii^—O3—Rb1A	6.8 (3)
O3^iii^—Rb1B—O3^ii^	90.9 (4)	As2—O3—Rb1B^i^	106.0 (2)
Rb1B^i^—Rb1B—O2^ii^	15.05 (2)	Rb1B—O3—Rb1B^i^	9.3 (5)
Rb1B^ii^—Rb1B—O2^ii^	52.10 (7)	Rb1B^ii^—O3—Rb1B^i^	10.1 (5)
O2^v^—Rb1B—O2^ii^	160.5 (4)	Rb1A—O3—Rb1B^i^	4.9 (2)
O2^i^—Rb1B—O2^ii^	112.87 (12)	As2^iv^—O4—Ga1^xx^	126.28 (6)
O3^iv^—Rb1B—O2^ii^	106.2 (3)	As2^iv^—O4—Rb1B^xxi^	120.71 (10)
O3—Rb1B—O2^ii^	107.8 (3)	Ga1^xx^—O4—Rb1B^xxi^	99.25 (5)
O3^v^—Rb1B—O2^ii^	118.1 (4)	As2^iv^—O4—Rb1A^xix^	121.85 (5)
O3^i^—Rb1B—O2^ii^	72.8 (2)	Ga1^xx^—O4—Rb1A^xix^	99.67 (4)
O2—Rb1B—O2^ii^	106.5 (4)	Rb1B^xxi^—O4—Rb1A^xix^	2.51 (13)
O2^iv^—Rb1B—O2^ii^	72.5 (2)	As2^iv^—O4—Rb1B^xix^	126.9 (3)
O3^iii^—Rb1B—O2^ii^	57.9 (3)	Ga1^xx^—O4—Rb1B^xix^	96.29 (16)
O3^ii^—Rb1B—O2^ii^	41.55 (19)	Rb1B^xxi^—O4—Rb1B^xix^	7.1 (3)
Rb1B^i^—Rb1B—O2^iii^	52.10 (12)	Rb1A^xix^—O4—Rb1B^xix^	5.2 (2)
Rb1B^ii^—Rb1B—O2^iii^	15.05 (5)	As2^iv^—O4—Rb1B^xxii^	117.91 (18)
O2^v^—Rb1B—O2^iii^	112.87 (12)	Ga1^xx^—O4—Rb1B^xxii^	103.25 (18)
O2^i^—Rb1B—O2^iii^	160.5 (4)	Rb1B^xxi^—O4—Rb1B^xxii^	4.1 (2)
O3^iv^—Rb1B—O2^iii^	107.8 (3)	Rb1A^xix^—O4—Rb1B^xxii^	3.99 (18)
O3—Rb1B—O2^iii^	106.2 (3)	Rb1B^xix^—O4—Rb1B^xxii^	9.0 (4)
O3^v^—Rb1B—O2^iii^	72.8 (2)	As2^iv^—O4—Rb1B	53.63 (19)
O3^i^—Rb1B—O2^iii^	118.1 (4)	Ga1^xx^—O4—Rb1B	72.96 (19)
O2—Rb1B—O2^iii^	72.5 (2)	Rb1B^xxi^—O4—Rb1B	134.20 (8)
O2^iv^—Rb1B—O2^iii^	106.5 (4)	Rb1A^xix^—O4—Rb1B	136.70 (6)
O3^iii^—Rb1B—O2^iii^	41.55 (19)	Rb1B^xix^—O4—Rb1B	139.53 (16)
O3^ii^—Rb1B—O2^iii^	57.9 (3)	Rb1B^xxii^—O4—Rb1B	135.79 (4)
O2^ii^—Rb1B—O2^iii^	48.4 (2)	As2^iv^—O4—Rb1B^i^	47.20 (15)
Rb1B^i^—Rb1B—O4^vi^	153.31 (5)	Ga1^xx^—O4—Rb1B^i^	80.09 (19)
Rb1B^ii^—Rb1B—O4^vi^	130.76 (15)	Rb1B^xxi^—O4—Rb1B^i^	130.0 (3)
O2^v^—Rb1B—O4^vi^	45.1 (2)	Rb1A^xix^—O4—Rb1B^i^	132.53 (17)
O2^i^—Rb1B—O4^vi^	53.4 (3)	Rb1B^xix^—O4—Rb1B^i^	136.12 (3)
O3^iv^—Rb1B—O4^vi^	98.0 (5)	Rb1B^xxii^—O4—Rb1B^i^	131.0 (3)
O3—Rb1B—O4^vi^	44.50 (19)	Rb1B—O4—Rb1B^i^	8.2 (4)
O3^v^—Rb1B—O4^vi^	93.3 (3)	As2^iv^—O4—Rb1A	50.21 (3)
O3^i^—Rb1B—O4^vi^	76.7 (3)	Ga1^xx^—O4—Rb1A	76.56 (3)
O2—Rb1B—O4^vi^	71.6 (2)	Rb1B^xxi^—O4—Rb1A	133.03 (14)
O2^iv^—Rb1B—O4^vi^	109.4 (4)	Rb1A^xix^—O4—Rb1A	135.53 (3)
O3^iii^—Rb1B—O4^vi^	157.2 (3)	Rb1B^xix^—O4—Rb1A	138.77 (15)
O3^ii^—Rb1B—O4^vi^	109.34 (9)	Rb1B^xxii^—O4—Rb1A	134.28 (7)
O2^ii^—Rb1B—O4^vi^	144.95 (3)	Rb1B—O4—Rb1A	3.8 (2)
O2^iii^—Rb1B—O4^vi^	144.11 (3)	Rb1B^i^—O4—Rb1A	4.5 (2)
Rb1B^i^—Rb1B—O4^vii^	130.76 (11)		

Symmetry codes: (i) −*y*, *x*−*y*, *z*; (ii) −*x*+*y*, −*x*, *z*; (iii) *x*−*y*, −*y*, −*z*+3/2; (iv) −*x*, −*x*+*y*, −*z*+3/2; (v) *y*, *x*, −*z*+3/2; (vi) *y*, *x*−1, −*z*+3/2; (vii) −*y*, *x*−*y*−1, *z*; (viii) *x*, *y*+1, *z*; (ix) −*x*+*y*+1, −*x*+1, *z*; (x) −*y*, *x*−*y*+1, *z*; (xi) *x*+1, *y*+1, *z*; (xii) *x*−*y*−1/3, *x*+1/3, −*z*+4/3; (xiii) *y*+2/3, −*x*+*y*+4/3, −*z*+4/3; (xiv) −*x*+2/3, −*y*+1/3, −*z*+4/3; (xv) *x*−1, *y*, *z*; (xvi) −*y*−1, *x*−*y*−1, *z*; (xvii) *x*−1, *y*−1, *z*; (xviii) −*x*+*y*−1, −*x*−1, *z*; (xix) *x*+1, *y*, *z*; (xx) *x*, *y*−1, *z*; (xxi) −*x*+*y*+1, −*x*, *z*; (xxii) −*y*+1, *x*−*y*, *z*.

### Rubidium digallium arsenic(V) hexakis[hydrogen arsenate(V)] (RbGa2AsHAsO46) Hydrogen-bond geometry (Å, º)

**Table:**

*D*—H···*A*	*D*—H	H···*A*	*D*···*A*	*D*—H···*A*
O3—H···O4^vi^	0.80 (3)	1.98 (3)	2.7314 (17)	158 (3)

Symmetry code: (vi) *y*, *x*−1, −*z*+3/2.

### Rubidium gallium bis[hydrogen arsenate(V)] (RbGaHAsO42) Crystal data

**Table:**

RbGa(HAsO_4_)_2_	*D*_x_ = 3.964 Mg m^−^^3^
*M**_r_* = 435.05	Mo *K*α radiation, λ = 0.71073 Å
Trigonal, *R*3*c*:*H*	Cell parameters from 2663 reflections
*a* = 8.385 (1) Å	θ = 2.3–30.0°
*c* = 53.880 (11) Å	µ = 19.42 mm^−^^1^
*V* = 3280.7 (10) Å^3^	*T* = 293 K
*Z* = 18	Hexagonal plate, colourless
*F*(000) = 3600	0.07 × 0.07 × 0.02 mm

### Rubidium gallium bis[hydrogen arsenate(V)] (RbGaHAsO42) Data collection

**Table:**

Nonius KappaCCD single-crystal four-circle diffractometer	1027 reflections with *I* > 2σ(*I*)
Radiation source: fine-focus sealed tube	*R*_int_ = 0.016
φ and ω scans	θ_max_ = 30.0°, θ_min_ = 2.3°
Absorption correction: multi-scan (SCALEPACK; Otwinowski *et al.*, 2003)	*h* = −11→11
*T*_min_ = 0.343, *T*_max_ = 0.697	*k* = −9→9
3896 measured reflections	*l* = −75→75
1079 independent reflections	

### Rubidium gallium bis[hydrogen arsenate(V)] (RbGaHAsO42) Refinement

**Table:**

Refinement on *F*^2^	Secondary atom site location: difference Fourier map
Least-squares matrix: full	Hydrogen site location: difference Fourier map
*R*[*F*^2^ > 2σ(*F*^2^)] = 0.016	All H-atom parameters refined
*wR*(*F*^2^) = 0.040	*w* = 1/[σ^2^(*F*_o_^2^) + (0.0168*P*)^2^ + 20.8962*P*] where *P* = (*F*_o_^2^ + 2*F*_c_^2^)/3
*S* = 1.11	(Δ/σ)_max_ = 0.014
1079 reflections	Δρ_max_ = 0.79 e Å^−^^3^
68 parameters	Δρ_min_ = −0.52 e Å^−^^3^
2 restraints	Extinction correction: SHELXL2016 (Sheldrick, 2015), Fc^*^=kFc[1+0.001xFc^2^λ^3^/sin(2θ)]^-1/4^
Primary atom site location: structure-invariant direct methods	Extinction coefficient: 0.000092 (13)

### Rubidium gallium bis[hydrogen arsenate(V)] (RbGaHAsO42) Special details

**Table:**

Geometry. All esds (except the esd in the dihedral angle between two l.s. planes) are estimated using the full covariance matrix. The cell esds are taken into account individually in the estimation of esds in distances, angles and torsion angles; correlations between esds in cell parameters are only used when they are defined by crystal symmetry. An approximate (isotropic) treatment of cell esds is used for estimating esds involving l.s. planes.

### Rubidium gallium bis[hydrogen arsenate(V)] (RbGaHAsO42) Fractional atomic coordinates and isotropic or equivalent isotropic displacement parameters (Å^2^)

**Table:**

	*x*	*y*	*z*	*U*_iso_*/*U*_eq_	Occ. (<1)
Rb1A	0.000000	0.000000	0.750000	0.0297 (12)	0.909 (3)
Rb1B	0.000000	−0.050 (5)	0.750000	0.011 (3)	0.0304 (9)
Rb2	0.000000	0.000000	0.66714 (2)	0.02912 (12)	
Ga1	0.333333	0.666667	0.75374 (2)	0.00733 (9)	
Ga2	0.333333	0.666667	0.666667	0.00817 (11)	
As	−0.43059 (3)	−0.39514 (3)	0.71280 (2)	0.00799 (7)	
O1	0.4536 (2)	−0.4402 (2)	0.68636 (3)	0.0159 (3)	
O2	−0.4459 (2)	−0.2541 (2)	0.73338 (3)	0.0106 (3)	
O3	−0.1974 (2)	−0.2813 (2)	0.70523 (3)	0.0178 (3)	
O4	0.4789 (2)	−0.1223 (2)	0.77582 (3)	0.0103 (3)	
H	−0.161 (5)	−0.354 (5)	0.7099 (6)	0.035 (10)*	

### Rubidium gallium bis[hydrogen arsenate(V)] (RbGaHAsO42) Atomic displacement parameters (Å^2^)

**Table:**

	*U*^11^	*U*^22^	*U*^33^	*U*^12^	*U*^13^	*U*^23^
Rb1A	0.0333 (16)	0.0333 (16)	0.0225 (9)	0.0166 (8)	0.000	0.000
Rb1B	0.023 (11)	0.012 (7)	0.004 (7)	0.011 (5)	−0.002 (4)	−0.001 (2)
Rb2	0.03481 (17)	0.03481 (17)	0.0177 (2)	0.01740 (9)	0.000	0.000
Ga1	0.00785 (12)	0.00785 (12)	0.00629 (16)	0.00393 (6)	0.000	0.000
Ga2	0.00943 (15)	0.00943 (15)	0.0057 (2)	0.00472 (8)	0.000	0.000
As	0.00991 (11)	0.00839 (10)	0.00729 (10)	0.00580 (8)	0.00068 (7)	0.00083 (7)
O1	0.0246 (8)	0.0212 (8)	0.0087 (6)	0.0164 (7)	−0.0050 (6)	−0.0011 (6)
O2	0.0105 (7)	0.0106 (6)	0.0104 (6)	0.0049 (5)	0.0026 (5)	−0.0016 (5)
O3	0.0129 (7)	0.0173 (8)	0.0257 (9)	0.0093 (7)	0.0085 (6)	0.0093 (6)
O4	0.0116 (6)	0.0092 (6)	0.0120 (6)	0.0066 (6)	−0.0019 (5)	−0.0038 (5)

### Rubidium gallium bis[hydrogen arsenate(V)] (RbGaHAsO42) Geometric parameters (Å, º)

**Table:**

Rb1A—Rb1B^i^	0.42 (4)	Rb2—O3^ii^	2.9347 (17)
Rb1A—Rb1B^ii^	0.42 (4)	Rb2—O1^vi^	3.3714 (16)
Rb1A—O3^iii^	3.197 (2)	Rb2—O1^vii^	3.3715 (16)
Rb1A—O3^iv^	3.197 (2)	Rb2—O1^viii^	3.3715 (17)
Rb1A—O3^ii^	3.197 (2)	Rb2—O4^ix^	3.4960 (16)
Rb1A—O3^i^	3.197 (2)	Rb2—O4^x^	3.4960 (17)
Rb1A—O3^v^	3.197 (2)	Rb2—O4^xi^	3.4960 (16)
Rb1A—O3	3.197 (2)	Rb2—O3^xii^	3.5327 (19)
Rb1A—O2^iv^	3.3698 (16)	Rb2—O3^xiii^	3.533 (2)
Rb1A—O2^ii^	3.3698 (16)	Rb2—O3^xiv^	3.5328 (19)
Rb1A—O2^v^	3.3699 (16)	Rb2—As^xiv^	3.7432 (5)
Rb1A—O2	3.3699 (16)	Rb2—As^xii^	3.7432 (5)
Rb1A—O2^iii^	3.3699 (15)	Rb2—As^xiii^	3.7432 (5)
Rb1A—O2^i^	3.3699 (15)	Ga1—O2^xv^	1.9596 (14)
Rb1A—As^iv^	4.0083 (5)	Ga1—O2^ii^	1.9597 (15)
Rb1B—Rb1B^i^	0.72 (7)	Ga1—O2^xvi^	1.9597 (15)
Rb1B—Rb1B^ii^	0.72 (7)	Ga1—O4^xvii^	1.9690 (15)
Rb1B—O3^iv^	3.019 (15)	Ga1—O4^i^	1.9690 (15)
Rb1B—O3	3.019 (15)	Ga1—O4^xviii^	1.9690 (15)
Rb1B—O2^v^	3.05 (3)	Ga2—O1^viii^	1.9625 (15)
Rb1B—O2^i^	3.05 (3)	Ga2—O1^xiv^	1.9625 (16)
Rb1B—O3^i^	3.161 (2)	Ga2—O1^xix^	1.9625 (15)
Rb1B—O3^v^	3.161 (2)	Ga2—O1^i^	1.9626 (15)
Rb1B—O2^iv^	3.363 (2)	Ga2—O1^xviii^	1.9626 (16)
Rb1B—O2	3.363 (2)	Ga2—O1^xvii^	1.9626 (15)
Rb1B—O3^iii^	3.47 (3)	As—O1^xx^	1.6576 (15)
Rb1B—O3^ii^	3.47 (3)	As—O2	1.6724 (15)
Rb2—O3	2.9346 (17)	As—O4^iv^	1.6805 (15)
Rb2—O3^i^	2.9347 (17)	As—O3	1.7417 (17)
			
Rb1B^i^—Rb1A—Rb1B^ii^	120.00 (15)	O1^vi^—Rb2—As^xii^	26.29 (3)
Rb1B^i^—Rb1A—O3^iii^	61.38 (3)	O1^vii^—Rb2—As^xii^	88.53 (3)
Rb1B^ii^—Rb1A—O3^iii^	81.43 (11)	O1^viii^—Rb2—As^xii^	105.88 (3)
Rb1B^i^—Rb1A—O3^iv^	81.43 (3)	O4^ix^—Rb2—As^xii^	26.56 (2)
Rb1B^ii^—Rb1A—O3^iv^	128.90 (5)	O4^x^—Rb2—As^xii^	54.15 (3)
O3^iii^—Rb1A—O3^iv^	69.26 (5)	O4^xi^—Rb2—As^xii^	71.43 (3)
Rb1B^i^—Rb1A—O3^ii^	81.43 (3)	O3^xii^—Rb2—As^xii^	27.50 (3)
Rb1B^ii^—Rb1A—O3^ii^	61.38 (9)	O3^xiii^—Rb2—As^xii^	63.12 (3)
O3^iii^—Rb1A—O3^ii^	102.21 (6)	O3^xiv^—Rb2—As^xii^	98.11 (3)
O3^iv^—Rb1A—O3^ii^	162.87 (7)	As^xiv^—Rb2—As^xii^	79.914 (13)
Rb1B^i^—Rb1A—O3^i^	61.38 (3)	O3—Rb2—As^xiii^	177.27 (4)
Rb1B^ii^—Rb1A—O3^i^	128.90 (5)	O3^i^—Rb2—As^xiii^	100.79 (4)
O3^iii^—Rb1A—O3^i^	122.76 (7)	O3^ii^—Rb2—As^xiii^	102.80 (4)
O3^iv^—Rb1A—O3^i^	102.21 (6)	O1^vi^—Rb2—As^xiii^	88.53 (3)
O3^ii^—Rb1A—O3^i^	69.26 (5)	O1^vii^—Rb2—As^xiii^	105.88 (3)
Rb1B^i^—Rb1A—O3^v^	128.90 (3)	O1^viii^—Rb2—As^xiii^	26.29 (3)
Rb1B^ii^—Rb1A—O3^v^	61.38 (9)	O4^ix^—Rb2—As^xiii^	54.15 (3)
O3^iii^—Rb1A—O3^v^	69.26 (5)	O4^x^—Rb2—As^xiii^	71.43 (3)
O3^iv^—Rb1A—O3^v^	69.26 (5)	O4^xi^—Rb2—As^xiii^	26.56 (3)
O3^ii^—Rb1A—O3^v^	122.76 (6)	O3^xii^—Rb2—As^xiii^	98.11 (3)
O3^i^—Rb1A—O3^v^	162.87 (6)	O3^xiii^—Rb2—As^xiii^	27.50 (3)
Rb1B^i^—Rb1A—O3	128.90 (3)	O3^xiv^—Rb2—As^xiii^	63.12 (3)
Rb1B^ii^—Rb1A—O3	81.43 (11)	As^xiv^—Rb2—As^xiii^	79.914 (13)
O3^iii^—Rb1A—O3	162.87 (6)	As^xii^—Rb2—As^xiii^	79.914 (13)
O3^iv^—Rb1A—O3	122.76 (6)	O2^xv^—Ga1—O2^ii^	91.72 (6)
O3^ii^—Rb1A—O3	69.26 (5)	O2^xv^—Ga1—O2^xvi^	91.72 (6)
O3^i^—Rb1A—O3	69.26 (5)	O2^ii^—Ga1—O2^xvi^	91.72 (6)
O3^v^—Rb1A—O3	102.21 (6)	O2^xv^—Ga1—O4^xvii^	92.25 (6)
Rb1B^i^—Rb1A—O2^iv^	37.49 (3)	O2^ii^—Ga1—O4^xvii^	175.98 (6)
Rb1B^ii^—Rb1A—O2^iv^	150.57 (13)	O2^xvi^—Ga1—O4^xvii^	88.72 (6)
O3^iii^—Rb1A—O2^iv^	70.43 (4)	O2^xv^—Ga1—O4^i^	88.72 (6)
O3^iv^—Rb1A—O2^iv^	48.04 (4)	O2^ii^—Ga1—O4^i^	92.25 (6)
O3^ii^—Rb1A—O2^iv^	115.61 (4)	O2^xvi^—Ga1—O4^i^	175.98 (7)
O3^i^—Rb1A—O2^iv^	64.84 (4)	O4^xvii^—Ga1—O4^i^	87.28 (6)
O3^v^—Rb1A—O2^iv^	113.71 (4)	O2^xv^—Ga1—O4^xviii^	175.98 (6)
O3—Rb1A—O2^iv^	126.47 (4)	O2^ii^—Ga1—O4^xviii^	88.71 (6)
Rb1B^i^—Rb1A—O2^ii^	37.49 (3)	O2^xvi^—Ga1—O4^xviii^	92.25 (6)
Rb1B^ii^—Rb1A—O2^ii^	85.56 (15)	O4^xvii^—Ga1—O4^xviii^	87.28 (7)
O3^iii^—Rb1A—O2^ii^	64.84 (4)	O4^i^—Ga1—O4^xviii^	87.28 (7)
O3^iv^—Rb1A—O2^ii^	115.61 (4)	O2^xv^—Ga1—Rb2^xxi^	124.04 (4)
O3^ii^—Rb1A—O2^ii^	48.04 (4)	O2^ii^—Ga1—Rb2^xxi^	124.04 (4)
O3^i^—Rb1A—O2^ii^	70.43 (4)	O2^xvi^—Ga1—Rb2^xxi^	124.04 (4)
O3^v^—Rb1A—O2^ii^	126.47 (4)	O4^xvii^—Ga1—Rb2^xxi^	52.83 (5)
O3—Rb1A—O2^ii^	113.71 (4)	O4^i^—Ga1—Rb2^xxi^	52.83 (5)
O2^iv^—Rb1A—O2^ii^	74.98 (5)	O4^xviii^—Ga1—Rb2^xxi^	52.83 (4)
Rb1B^i^—Rb1A—O2^v^	150.57 (3)	O2^xv^—Ga1—Rb1A^xvii^	32.81 (4)
Rb1B^ii^—Rb1A—O2^v^	85.56 (15)	O2^ii^—Ga1—Rb1A^xvii^	105.67 (4)
O3^iii^—Rb1A—O2^v^	113.71 (4)	O2^xvi^—Ga1—Rb1A^xvii^	120.04 (5)
O3^iv^—Rb1A—O2^v^	70.43 (4)	O4^xvii^—Ga1—Rb1A^xvii^	77.51 (4)
O3^ii^—Rb1A—O2^v^	126.47 (4)	O4^i^—Ga1—Rb1A^xvii^	59.26 (4)
O3^i^—Rb1A—O2^v^	115.61 (4)	O4^xviii^—Ga1—Rb1A^xvii^	143.41 (5)
O3^v^—Rb1A—O2^v^	48.04 (4)	Rb2^xxi^—Ga1—Rb1A^xvii^	92.384 (4)
O3—Rb1A—O2^v^	64.84 (4)	O2^xv^—Ga1—Rb1A	120.04 (5)
O2^iv^—Rb1A—O2^v^	113.21 (2)	O2^ii^—Ga1—Rb1A	32.81 (4)
O2^ii^—Rb1A—O2^v^	171.11 (5)	O2^xvi^—Ga1—Rb1A	105.67 (4)
Rb1B^i^—Rb1A—O2	150.57 (3)	O4^xvii^—Ga1—Rb1A	143.41 (5)
Rb1B^ii^—Rb1A—O2	37.49 (14)	O4^i^—Ga1—Rb1A	77.51 (4)
O3^iii^—Rb1A—O2	115.61 (4)	O4^xviii^—Ga1—Rb1A	59.26 (5)
O3^iv^—Rb1A—O2	126.47 (4)	Rb2^xxi^—Ga1—Rb1A	92.384 (4)
O3^ii^—Rb1A—O2	70.43 (4)	Rb1A^xvii^—Ga1—Rb1A	119.828 (1)
O3^i^—Rb1A—O2	113.71 (4)	O2^xv^—Ga1—Rb1A^xvi^	105.67 (5)
O3^v^—Rb1A—O2	64.84 (4)	O2^ii^—Ga1—Rb1A^xvi^	120.04 (5)
O3—Rb1A—O2	48.04 (4)	O2^xvi^—Ga1—Rb1A^xvi^	32.81 (4)
O2^iv^—Rb1A—O2	171.11 (5)	O4^xvii^—Ga1—Rb1A^xvi^	59.26 (4)
O2^ii^—Rb1A—O2	113.21 (2)	O4^i^—Ga1—Rb1A^xvi^	143.41 (5)
O2^v^—Rb1A—O2	58.86 (5)	O4^xviii^—Ga1—Rb1A^xvi^	77.51 (5)
Rb1B^i^—Rb1A—O2^iii^	85.56 (3)	Rb2^xxi^—Ga1—Rb1A^xvi^	92.384 (4)
Rb1B^ii^—Rb1A—O2^iii^	37.49 (14)	Rb1A^xvii^—Ga1—Rb1A^xvi^	119.828 (1)
O3^iii^—Rb1A—O2^iii^	48.04 (4)	Rb1A—Ga1—Rb1A^xvi^	119.828 (1)
O3^iv^—Rb1A—O2^iii^	113.71 (4)	O1^viii^—Ga2—O1^xiv^	93.53 (6)
O3^ii^—Rb1A—O2^iii^	64.84 (4)	O1^viii^—Ga2—O1^xix^	93.53 (6)
O3^i^—Rb1A—O2^iii^	126.47 (4)	O1^xiv^—Ga2—O1^xix^	93.53 (6)
O3^v^—Rb1A—O2^iii^	70.43 (4)	O1^viii^—Ga2—O1^i^	180.0
O3—Rb1A—O2^iii^	115.61 (4)	O1^xiv^—Ga2—O1^i^	86.48 (6)
O2^iv^—Rb1A—O2^iii^	113.21 (2)	O1^xix^—Ga2—O1^i^	86.48 (6)
O2^ii^—Rb1A—O2^iii^	58.87 (5)	O1^viii^—Ga2—O1^xviii^	86.48 (6)
O2^v^—Rb1A—O2^iii^	113.21 (2)	O1^xiv^—Ga2—O1^xviii^	180.0
O2—Rb1A—O2^iii^	74.98 (5)	O1^xix^—Ga2—O1^xviii^	86.48 (6)
Rb1B^i^—Rb1A—O2^i^	85.56 (3)	O1^i^—Ga2—O1^xviii^	93.52 (6)
Rb1B^ii^—Rb1A—O2^i^	150.57 (14)	O1^viii^—Ga2—O1^xvii^	86.48 (6)
O3^iii^—Rb1A—O2^i^	126.47 (4)	O1^xiv^—Ga2—O1^xvii^	86.48 (6)
O3^iv^—Rb1A—O2^i^	64.84 (4)	O1^xix^—Ga2—O1^xvii^	180.0
O3^ii^—Rb1A—O2^i^	113.71 (4)	O1^i^—Ga2—O1^xvii^	93.52 (6)
O3^i^—Rb1A—O2^i^	48.04 (4)	O1^xviii^—Ga2—O1^xvii^	93.52 (6)
O3^v^—Rb1A—O2^i^	115.61 (4)	O1^viii^—Ga2—Rb2^xix^	63.39 (5)
O3—Rb1A—O2^i^	70.43 (4)	O1^xiv^—Ga2—Rb2^xix^	66.53 (5)
O2^iv^—Rb1A—O2^i^	58.87 (5)	O1^xix^—Ga2—Rb2^xix^	146.92 (4)
O2^ii^—Rb1A—O2^i^	113.21 (2)	O1^i^—Ga2—Rb2^xix^	116.61 (5)
O2^v^—Rb1A—O2^i^	74.98 (5)	O1^xviii^—Ga2—Rb2^xix^	113.47 (5)
O2—Rb1A—O2^i^	113.21 (2)	O1^xvii^—Ga2—Rb2^xix^	33.08 (4)
O2^iii^—Rb1A—O2^i^	171.11 (6)	O1^viii^—Ga2—Rb2^xvii^	116.62 (5)
Rb1B^i^—Rb1A—As^iv^	60.827 (6)	O1^xiv^—Ga2—Rb2^xvii^	113.48 (5)
Rb1B^ii^—Rb1A—As^iv^	149.73 (2)	O1^xix^—Ga2—Rb2^xvii^	33.08 (4)
O3^iii^—Rb1A—As^iv^	73.16 (3)	O1^i^—Ga2—Rb2^xvii^	63.38 (5)
O3^iv^—Rb1A—As^iv^	24.86 (3)	O1^xviii^—Ga2—Rb2^xvii^	66.52 (5)
O3^ii^—Rb1A—As^iv^	139.65 (3)	O1^xvii^—Ga2—Rb2^xvii^	146.92 (4)
O3^i^—Rb1A—As^iv^	79.79 (3)	Rb2^xix^—Ga2—Rb2^xvii^	180.0
O3^v^—Rb1A—As^iv^	93.74 (3)	O1^viii^—Ga2—Rb2^xvi^	113.48 (5)
O3—Rb1A—As^iv^	123.16 (3)	O1^xiv^—Ga2—Rb2^xvi^	33.08 (4)
O2^iv^—Rb1A—As^iv^	24.28 (2)	O1^xix^—Ga2—Rb2^xvi^	116.62 (6)
O2^ii^—Rb1A—As^iv^	97.93 (3)	O1^i^—Ga2—Rb2^xvi^	66.52 (5)
O2^v^—Rb1A—As^iv^	89.76 (3)	O1^xviii^—Ga2—Rb2^xvi^	146.92 (4)
O2—Rb1A—As^iv^	148.57 (3)	O1^xvii^—Ga2—Rb2^xvi^	63.38 (6)
O2^iii^—Rb1A—As^iv^	121.15 (3)	Rb2^xix^—Ga2—Rb2^xvi^	60.0
O2^i^—Rb1A—As^iv^	53.64 (3)	Rb2^xvii^—Ga2—Rb2^xvi^	120.0
Rb1B^i^—Rb1B—Rb1B^ii^	60.00 (4)	O1^viii^—Ga2—Rb2	33.08 (5)
Rb1B^i^—Rb1B—O3^iv^	94.7 (6)	O1^xiv^—Ga2—Rb2	116.62 (5)
Rb1B^ii^—Rb1B—O3^iv^	123.8 (6)	O1^xix^—Ga2—Rb2	113.48 (5)
Rb1B^i^—Rb1B—O3	123.8 (6)	O1^i^—Ga2—Rb2	146.92 (5)
Rb1B^ii^—Rb1B—O3	94.7 (7)	O1^xviii^—Ga2—Rb2	63.38 (5)
O3^iv^—Rb1B—O3	136.7 (14)	O1^xvii^—Ga2—Rb2	66.52 (5)
Rb1B^i^—Rb1B—O2^v^	160.6 (4)	Rb2^xix^—Ga2—Rb2	60.0
Rb1B^ii^—Rb1B—O2^v^	109.8 (6)	Rb2^xvii^—Ga2—Rb2	120.0
O3^iv^—Rb1B—O2^v^	77.3 (7)	Rb2^xvi^—Ga2—Rb2	120.0
O3—Rb1B—O2^v^	71.0 (6)	O1^viii^—Ga2—Rb2^xxii^	66.52 (5)
Rb1B^i^—Rb1B—O2^i^	109.8 (5)	O1^xiv^—Ga2—Rb2^xxii^	146.92 (4)
Rb1B^ii^—Rb1B—O2^i^	160.6 (3)	O1^xix^—Ga2—Rb2^xxii^	63.38 (6)
O3^iv^—Rb1B—O2^i^	71.0 (6)	O1^i^—Ga2—Rb2^xxii^	113.47 (5)
O3—Rb1B—O2^i^	77.3 (7)	O1^xviii^—Ga2—Rb2^xxii^	33.08 (4)
O2^v^—Rb1B—O2^i^	84.6 (10)	O1^xvii^—Ga2—Rb2^xxii^	116.62 (6)
Rb1B^i^—Rb1B—O3^i^	72.1 (6)	Rb2^xix^—Ga2—Rb2^xxii^	120.0
Rb1B^ii^—Rb1B—O3^i^	109.8 (6)	Rb2^xvii^—Ga2—Rb2^xxii^	60.0
O3^iv^—Rb1B—O3^i^	107.2 (4)	Rb2^xvi^—Ga2—Rb2^xxii^	180.0
O3—Rb1B—O3^i^	72.0 (2)	Rb2—Ga2—Rb2^xxii^	60.0
O2^v^—Rb1B—O3^i^	127.0 (11)	O1^viii^—Ga2—Rb2^xxiii^	146.92 (5)
O2^i^—Rb1B—O3^i^	51.0 (2)	O1^xiv^—Ga2—Rb2^xxiii^	63.38 (5)
Rb1B^i^—Rb1B—O3^v^	109.8 (7)	O1^xix^—Ga2—Rb2^xxiii^	66.52 (5)
Rb1B^ii^—Rb1B—O3^v^	72.1 (7)	O1^i^—Ga2—Rb2^xxiii^	33.08 (5)
O3^iv^—Rb1B—O3^v^	72.0 (2)	O1^xviii^—Ga2—Rb2^xxiii^	116.61 (5)
O3—Rb1B—O3^v^	107.2 (4)	O1^xvii^—Ga2—Rb2^xxiii^	113.47 (5)
O2^v^—Rb1B—O3^v^	51.0 (2)	Rb2^xix^—Ga2—Rb2^xxiii^	120.0
O2^i^—Rb1B—O3^v^	127.0 (11)	Rb2^xvii^—Ga2—Rb2^xxiii^	60.0
O3^i^—Rb1B—O3^v^	177.9 (14)	Rb2^xvi^—Ga2—Rb2^xxiii^	60.0
Rb1B^i^—Rb1B—O2^iv^	58.5 (7)	Rb2—Ga2—Rb2^xxiii^	180.0
Rb1B^ii^—Rb1B—O2^iv^	116.2 (6)	Rb2^xxii^—Ga2—Rb2^xxiii^	119.997 (1)
O3^iv^—Rb1B—O2^iv^	49.25 (8)	O1^xx^—As—O2	119.20 (8)
O3—Rb1B—O2^iv^	133.4 (5)	O1^xx^—As—O4^iv^	105.45 (8)
O2^v^—Rb1B—O2^iv^	122.6 (9)	O2—As—O4^iv^	114.88 (7)
O2^i^—Rb1B—O2^iv^	62.0 (3)	O1^xx^—As—O3	107.00 (9)
O3^i^—Rb1B—O2^iv^	65.28 (4)	O2—As—O3	103.30 (8)
O3^v^—Rb1B—O2^iv^	114.83 (5)	O4^iv^—As—O3	106.04 (8)
Rb1B^i^—Rb1B—O2	116.2 (7)	O1^xx^—As—Rb2^xii^	64.25 (6)
Rb1B^ii^—Rb1B—O2	58.5 (8)	O2—As—Rb2^xii^	172.80 (5)
O3^iv^—Rb1B—O2	133.4 (5)	O4^iv^—As—Rb2^xii^	68.49 (5)
O3—Rb1B—O2	49.25 (8)	O3—As—Rb2^xii^	69.51 (6)
O2^v^—Rb1B—O2	62.0 (3)	O1^xx^—As—Rb1B^ii^	142.0 (2)
O2^i^—Rb1B—O2	122.6 (9)	O2—As—Rb1B^ii^	50.6 (6)
O3^i^—Rb1B—O2	114.83 (5)	O4^iv^—As—Rb1B^ii^	111.5 (3)
O3^v^—Rb1B—O2	65.28 (4)	O3—As—Rb1B^ii^	54.9 (5)
O2^iv^—Rb1B—O2	174.7 (13)	Rb2^xii^—As—Rb1B^ii^	122.5 (5)
Rb1B^i^—Rb1B—O3^iii^	46.2 (3)	O1^xx^—As—Rb1B	146.8 (4)
Rb1B^ii^—Rb1B—O3^iii^	58.9 (3)	O2—As—Rb1B	60.0 (4)
O3^iv^—Rb1B—O3^iii^	67.6 (2)	O4^iv^—As—Rb1B	103.6 (5)
O3—Rb1B—O3^iii^	153.6 (10)	O3—As—Rb1B	48.68 (19)
O2^v^—Rb1B—O3^iii^	114.75 (4)	Rb2^xii^—As—Rb1B	113.4 (4)
O2^i^—Rb1B—O3^iii^	127.90 (5)	Rb1B^ii^—As—Rb1B	10.8 (11)
O3^i^—Rb1B—O3^iii^	115.4 (7)	O1^xx^—As—Rb1A	143.22 (6)
O3^v^—Rb1B—O3^iii^	66.2 (3)	O2—As—Rb1A	55.94 (5)
O2^iv^—Rb1B—O3^iii^	67.3 (3)	O4^iv^—As—Rb1A	108.77 (5)
O2—Rb1B—O3^iii^	108.7 (7)	O3—As—Rb1A	50.50 (6)
Rb1B^i^—Rb1B—O3^ii^	58.9 (4)	Rb2^xii^—As—Rb1A	117.262 (8)
Rb1B^ii^—Rb1B—O3^ii^	46.2 (3)	Rb1B^ii^—As—Rb1A	5.5 (5)
O3^iv^—Rb1B—O3^ii^	153.6 (10)	Rb1B—As—Rb1A	5.7 (6)
O3—Rb1B—O3^ii^	67.6 (2)	O1^xx^—As—Rb2	81.53 (7)
O2^v^—Rb1B—O3^ii^	127.90 (5)	O2—As—Rb2	99.68 (5)
O2^i^—Rb1B—O3^ii^	114.75 (4)	O4^iv^—As—Rb2	133.31 (5)
O3^i^—Rb1B—O3^ii^	66.2 (3)	O3—As—Rb2	32.31 (6)
O3^v^—Rb1B—O3^ii^	115.4 (7)	Rb2^xii^—As—Rb2	74.193 (8)
O2^iv^—Rb1B—O3^ii^	108.7 (7)	Rb1B^ii^—As—Rb2	67.2 (2)
O2—Rb1B—O3^ii^	67.3 (3)	Rb1B—As—Rb2	66.79 (16)
O3^iii^—Rb1B—O3^ii^	91.5 (9)	Rb1A—As—Rb2	65.327 (14)
O3—Rb2—O3^i^	76.48 (6)	O1^xx^—As—Rb1A^xxiv^	87.84 (7)
O3—Rb2—O3^ii^	76.48 (6)	O2—As—Rb1A^xxiv^	94.28 (5)
O3^i^—Rb2—O3^ii^	76.48 (6)	O4^iv^—As—Rb1A^xxiv^	40.78 (5)
O3—Rb2—O1^vi^	91.00 (4)	O3—As—Rb1A^xxiv^	146.81 (6)
O3^i^—Rb2—O1^vi^	76.80 (4)	Rb2^xii^—As—Rb1A^xxiv^	92.146 (8)
O3^ii^—Rb2—O1^vi^	152.49 (5)	Rb1B^ii^—As—Rb1A^xxiv^	126.24 (14)
O3—Rb2—O1^vii^	76.80 (5)	Rb1B—As—Rb1A^xxiv^	125.1 (3)
O3^i^—Rb2—O1^vii^	152.49 (5)	Rb1A—As—Rb1A^xxiv^	127.415 (12)
O3^ii^—Rb2—O1^vii^	91.00 (4)	Rb2—As—Rb1A^xxiv^	165.357 (7)
O1^vi^—Rb2—O1^vii^	110.14 (3)	O1^xx^—As—Rb2^xxiv^	42.87 (6)
O3—Rb2—O1^viii^	152.49 (5)	O2—As—Rb2^xxiv^	127.54 (5)
O3^i^—Rb2—O1^viii^	91.00 (5)	O4^iv^—As—Rb2^xxiv^	64.16 (5)
O3^ii^—Rb2—O1^viii^	76.80 (5)	O3—As—Rb2^xxiv^	128.20 (6)
O1^vi^—Rb2—O1^viii^	110.14 (3)	Rb2^xii^—As—Rb2^xxiv^	59.507 (5)
O1^vii^—Rb2—O1^viii^	110.13 (2)	Rb1B^ii^—As—Rb2^xxiv^	174.80 (5)
O3—Rb2—O4^ix^	126.84 (4)	Rb1B—As—Rb2^xxiv^	167.1 (6)
O3^i^—Rb2—O4^ix^	111.15 (5)	Rb1A—As—Rb2^xxiv^	172.736 (5)
O3^ii^—Rb2—O4^ix^	156.15 (4)	Rb2—As—Rb2^xxiv^	117.769 (15)
O1^vi^—Rb2—O4^ix^	45.47 (4)	Rb1A^xxiv^—As—Rb2^xxiv^	48.647 (11)
O1^vii^—Rb2—O4^ix^	90.21 (4)	As^xxv^—O1—Ga2^xxvi^	137.45 (10)
O1^viii^—Rb2—O4^ix^	80.43 (4)	As^xxv^—O1—Rb2^vi^	89.47 (6)
O3—Rb2—O4^x^	111.15 (5)	Ga2^xxvi^—O1—Rb2^vi^	128.38 (6)
O3^i^—Rb2—O4^x^	156.15 (4)	As^xxv^—O1—Rb2^xxv^	76.24 (6)
O3^ii^—Rb2—O4^x^	126.84 (4)	Ga2^xxvi^—O1—Rb2^xxv^	92.73 (6)
O1^vi^—Rb2—O4^x^	80.43 (4)	Rb2^vi^—O1—Rb2^xxv^	76.74 (3)
O1^vii^—Rb2—O4^x^	45.46 (4)	As^xxv^—O1—Rb2^xxvi^	122.41 (7)
O1^viii^—Rb2—O4^x^	90.21 (4)	Ga2^xxvi^—O1—Rb2^xxvi^	89.56 (6)
O4^ix^—Rb2—O4^x^	45.74 (4)	Rb2^vi^—O1—Rb2^xxvi^	75.20 (3)
O3—Rb2—O4^xi^	156.15 (5)	Rb2^xxv^—O1—Rb2^xxvi^	145.76 (4)
O3^i^—Rb2—O4^xi^	126.84 (5)	As—O2—Ga1^xxiv^	121.66 (8)
O3^ii^—Rb2—O4^xi^	111.15 (5)	As—O2—Rb1B^ii^	104.3 (5)
O1^vi^—Rb2—O4^xi^	90.21 (4)	Ga1^xxiv^—O2—Rb1B^ii^	125.9 (3)
O1^vii^—Rb2—O4^xi^	80.43 (4)	As—O2—Rb1B	94.4 (5)
O1^viii^—Rb2—O4^xi^	45.46 (4)	Ga1^xxiv^—O2—Rb1B	130.14 (12)
O4^ix^—Rb2—O4^xi^	45.74 (4)	Rb1B^ii^—O2—Rb1B	11.7 (11)
O4^x^—Rb2—O4^xi^	45.74 (4)	As—O2—Rb1A	99.78 (6)
O3—Rb2—O3^xii^	83.50 (5)	Ga1^xxiv^—O2—Rb1A	128.83 (6)
O3^i^—Rb2—O3^xii^	119.25 (6)	Rb1B^ii^—O2—Rb1A	4.8 (5)
O3^ii^—Rb2—O3^xii^	150.88 (6)	Rb1B—O2—Rb1A	7.1 (7)
O1^vi^—Rb2—O3^xii^	46.57 (4)	As—O2—Rb2	60.35 (4)
O1^vii^—Rb2—O3^xii^	63.66 (4)	Ga1^xxiv^—O2—Rb2	162.84 (6)
O1^viii^—Rb2—O3^xii^	123.76 (4)	Rb1B^ii^—O2—Rb2	64.9 (2)
O4^ix^—Rb2—O3^xii^	45.78 (4)	Rb1B—O2—Rb2	63.47 (7)
O4^x^—Rb2—O3^xii^	43.39 (4)	Rb1A—O2—Rb2	63.10 (2)
O4^xi^—Rb2—O3^xii^	80.11 (4)	As—O3—Rb2	129.19 (8)
O3—Rb2—O3^xiii^	150.89 (6)	As—O3—Rb1B	105.64 (12)
O3^i^—Rb2—O3^xiii^	83.50 (5)	Rb2—O3—Rb1B	97.7 (5)
O3^ii^—Rb2—O3^xiii^	119.25 (6)	As—O3—Rb1B^ii^	98.2 (6)
O1^vi^—Rb2—O3^xiii^	63.66 (4)	Rb2—O3—Rb1B^ii^	94.64 (16)
O1^vii^—Rb2—O3^xiii^	123.75 (4)	Rb1B—O3—Rb1B^ii^	13.2 (13)
O1^viii^—Rb2—O3^xiii^	46.57 (4)	As—O3—Rb1A	104.65 (8)
O4^ix^—Rb2—O3^xiii^	43.39 (4)	Rb2—O3—Rb1A	93.37 (5)
O4^x^—Rb2—O3^xiii^	80.11 (4)	Rb1B—O3—Rb1A	7.0 (7)
O4^xi^—Rb2—O3^xiii^	45.78 (4)	Rb1B^ii^—O3—Rb1A	7.5 (7)
O3^xii^—Rb2—O3^xiii^	88.19 (4)	As—O3—Rb1B^i^	109.5 (4)
O3—Rb2—O3^xiv^	119.25 (6)	Rb2—O3—Rb1B^i^	88.4 (5)
O3^i^—Rb2—O3^xiv^	150.88 (6)	Rb1B—O3—Rb1B^i^	10.0 (9)
O3^ii^—Rb2—O3^xiv^	83.50 (5)	Rb1B^ii^—O3—Rb1B^i^	11.3 (10)
O1^vi^—Rb2—O3^xiv^	123.76 (4)	Rb1A—O3—Rb1B^i^	5.4 (5)
O1^vii^—Rb2—O3^xiv^	46.57 (4)	As—O3—Rb2^xii^	82.99 (7)
O1^viii^—Rb2—O3^xiv^	63.66 (4)	Rb2—O3—Rb2^xii^	96.50 (5)
O4^ix^—Rb2—O3^xiv^	80.11 (4)	Rb1B—O3—Rb2^xii^	152.4 (7)
O4^x^—Rb2—O3^xiv^	45.78 (4)	Rb1B^ii^—O3—Rb2^xii^	164.5 (4)
O4^xi^—Rb2—O3^xiv^	43.38 (4)	Rb1A—O3—Rb2^xii^	159.28 (6)
O3^xii^—Rb2—O3^xiv^	88.19 (4)	Rb1B^i^—O3—Rb2^xii^	159.23 (8)
O3^xiii^—Rb2—O3^xiv^	88.19 (4)	As^iv^—O4—Ga1^xxvi^	130.00 (8)
O3—Rb2—As^xiv^	102.80 (4)	As^iv^—O4—Rb2^xxvii^	84.95 (5)
O3^i^—Rb2—As^xiv^	177.27 (4)	Ga1^xxvi^—O4—Rb2^xxvii^	100.50 (6)
O3^ii^—Rb2—As^xiv^	100.79 (4)	As^iv^—O4—Rb1A^xxv^	124.06 (6)
O1^vi^—Rb2—As^xiv^	105.88 (3)	Ga1^xxvi^—O4—Rb1A^xxv^	96.95 (5)
O1^vii^—Rb2—As^xiv^	26.29 (3)	Rb2^xxvii^—O4—Rb1A^xxv^	118.51 (4)
O1^viii^—Rb2—As^xiv^	88.53 (3)	As^iv^—O4—Rb1A	51.95 (4)
O4^ix^—Rb2—As^xiv^	71.43 (3)	Ga1^xxvi^—O4—Rb1A	78.99 (4)
O4^x^—Rb2—As^xiv^	26.56 (2)	Rb2^xxvii^—O4—Rb1A	104.39 (3)
O4^xi^—Rb2—As^xiv^	54.15 (3)	Rb1A^xxv^—O4—Rb1A	136.81 (4)
O3^xii^—Rb2—As^xiv^	63.12 (3)	As^iv^—O4—Rb2^xxviii^	98.27 (5)
O3^xiii^—Rb2—As^xiv^	98.10 (3)	Ga1^xxvi^—O4—Rb2^xxviii^	129.76 (6)
O3^xiv^—Rb2—As^xiv^	27.50 (3)	Rb2^xxvii^—O4—Rb2^xxviii^	66.64 (2)
O3—Rb2—As^xii^	100.79 (4)	Rb1A^xxv^—O4—Rb2^xxviii^	57.20 (2)
O3^i^—Rb2—As^xii^	102.80 (4)	Rb1A—O4—Rb2^xxviii^	150.18 (3)
O3^ii^—Rb2—As^xii^	177.27 (4)		

Symmetry codes: (i) −*y*, *x*−*y*, *z*; (ii) −*x*+*y*, −*x*, *z*; (iii) *x*−*y*, −*y*, −*z*+3/2; (iv) −*x*, −*x*+*y*, −*z*+3/2; (v) *y*, *x*, −*z*+3/2; (vi) −*x*+2/3, −*y*−2/3, −*z*+4/3; (vii) *x*−*y*−4/3, *x*−2/3, −*z*+4/3; (viii) *y*+2/3, −*x*+*y*+4/3, −*z*+4/3; (ix) *x*−1/3, *x*−*y*−2/3, *z*−1/6; (x) −*y*−1/3, −*x*+1/3, *z*−1/6; (xi) −*x*+*y*+2/3, *y*+1/3, *z*−1/6; (xii) −*x*−1/3, −*y*−2/3, −*z*+4/3; (xiii) *y*+2/3, −*x*+*y*+1/3, −*z*+4/3; (xiv) *x*−*y*−1/3, *x*+1/3, −*z*+4/3; (xv) −*y*, *x*−*y*+1, *z*; (xvi) *x*+1, *y*+1, *z*; (xvii) *x*, *y*+1, *z*; (xviii) −*x*+*y*+1, −*x*+1, *z*; (xix) −*x*+2/3, −*y*+1/3, −*z*+4/3; (xx) *x*−1, *y*, *z*; (xxi) −*y*+1/3, −*x*+2/3, *z*+1/6; (xxii) −*x*−1/3, −*y*+1/3, −*z*+4/3; (xxiii) −*x*+2/3, −*y*+4/3, −*z*+4/3; (xxiv) *x*−1, *y*−1, *z*; (xxv) *x*+1, *y*, *z*; (xxvi) *x*, *y*−1, *z*; (xxvii) −*y*+1/3, −*x*−1/3, *z*+1/6; (xxviii) *y*+1, *x*, −*z*+3/2.

### Rubidium gallium bis[hydrogen arsenate(V)] (RbGaHAsO42) Hydrogen-bond geometry (Å, º)

**Table:**

*D*—H···*A*	*D*—H	H···*A*	*D*···*A*	*D*—H···*A*
O3—H···O4^xxix^	0.85 (3)	1.76 (3)	2.598 (2)	168 (4)

Symmetry code: (xxix) *y*, *x*−1, −*z*+3/2.
